# Readmission and mortality among children requiring long-term mechanical ventilation via tracheostomy: a systematic review

**DOI:** 10.1186/s12890-025-03818-3

**Published:** 2025-08-11

**Authors:** Lindsey Scheller, Karley Mariano, Sandra Staveski, Sandra Weiss, Abbey Alkon, Christopher J. Russell, Leia Casey, Yoshimi Fukuoka

**Affiliations:** 1 School of Nursing, University of California San Francisco, 490 Illinois St., San Francisco, CA 94143 USA; 2 Stanford University, Stanford, CA USA

**Keywords:** Tracheostomy, Long-term mechanical ventilation, Children, Readmission, Hospitalization, Mortality, Systematic review

## Abstract

**Background:**

Home environments improve quality of life and reduce infections for children on long-term mechanical ventilation via tracheostomy (LTMV-T). However, unexpected hospital readmissions and death remain significant concerns. Existing systematic reviews have not fully examined risk factors for readmission and mortality. This review examines modifiable and non-modifiable risk factors associated with readmission and mortality in infants, children, and adolescents on LTMV-T.

**Methods:**

Five databases (PubMed, CINAHL, Web of Science, Embase, and Epistemonikos) were searched from inception to 2024. All quantitative study designs examining risk factors associated with readmission and/or mortality in children less than 21 years of age on LTMV-T were included. Articles were limited to peer-reviewed journals and the English language. Covidence software was used for data management, study screening, and data extraction. Each abstract was reviewed by two independent reviewers and discrepancies were resolved by a third. The Joanna Briggs Institute critical appraisal tools were used to assess risk of bias in individual studies.

**Results:**

Twenty-six studies examined cohorts of children on LTMV-T from 1980 to 2023. Studies were primarily retrospective cohorts, with sample sizes ranging from 27 to 8,009 children. Most studies reported that at least 50% of readmissions occurred within the first two years post-discharge and respiratory-related issues accounted for 30–75% of readmissions. Mortality within the first-year post-discharge varied as low as 0% to as high as 16%. Few studies examined socioenvironmental risk factors or those specific to LTMV-T populations, conducting analyses primarily on tracheostomy-only and/or LTMV-T cohorts. Risk factors for readmission and mortality included age, lower income, discharge disposition, chronic conditions, lack of respiratory physiotherapy (cough assist, percussions), gastrostomy tube, and lower birth weight. Risk of bias ranged from low to moderate due to unclear outcome measures and analyses that did not address potential confounders.

**Conclusions:**

Readmissions are common occurrences among children on LTMV-T with considerable risk of mortality, especially within the first two-years post-discharge. Risk factors identified were predominately clinical and demographic characteristics that can inform risk assessments and targeted interventions. Future studies should further explore socioenvironmental factors such as social determinants of health among LTMV-T specific populations.

**Trial registration:**

International Prospective Register of Systematic Reviews ID: CRD42024492773.

**Supplementary Information:**

The online version contains supplementary material available at 10.1186/s12890-025-03818-3.

## Introduction

During the last two decades, high-income countries have reported an increase in both non-invasive ventilation and long-term mechanical ventilation via tracheostomy (LTMV-T) prevalence ranging from 0.2 to 8.0 per 100,000 children [1–6]. Advancements in technology and clinical care have enabled children who previously relied on prolonged hospitalizations and institutionalization in long-term care facilities for support to receive LTMV-T in their homes. Compared to the hospital, being at home provides children on LTMV-T with better quality of life (QOL), development, autonomy, and fewer infections [7–10]. Home-based care is the most cost-effective setting compared to hospitals or long-term care facilities [11]. In the United States, hospitals have reported a notable 55% to 62% increase in the number of hospital discharges on LTMV-T over 6- and 10-year study periods [5, 12]. Comparable patterns have been observed worldwide, with the United Kingdom witnessing a 30-fold rise in the number of children requiring LTMV-T from 1999 to 2010 [13]. With a rise in the number of children on LTMV-T at home, ensuring optimal health outcomes after discharge is important.

In the United States, readmission and mortality rates for children on LTMV-T are higher than the general pediatric population [14–18]. Readmission following a previous hospital admission is a national quality metric of care measured by hospitals and may indicate challenges in managing the child’s health in the home or community setting [19]. Hospital readmissions decrease QOL, disrupt the child’s daily life and development, increase the risk of mortality, and place strain on family functioning and finances [20–24]. With little improvement in readmission and mortality in recent decades, identifying and understanding risk factors associated with readmission and mortality in children on LTMV-T is important to prevent these outcomes [18, 25].

Results from a systematic review identified higher proportion of mortality among children on LTMV-T compared to children on non-invasive mechanical ventilation [18]. However, discussions on risk factors associated with mortality were limited and focused on underlying diagnoses such as central hypoventilation, cardiac disease, and pulmonary disease or airway abnormalities as risk factors. To the best of our knowledge, no reviews have fully described readmissions and mortality or examined risk factors specifically among children on LTMV-T. Moreover, no review has been published on summarizing modifiable/socioenvironmental risk factors (e.g., access to healthcare, caregiver psychosocial support, home nursing) that could be used for intervention development to provide support in the home setting. Investigating modifiable and non-modifiable risk factors will help identify groups with varying clinical and social needs, guide tailored intervention development across care settings, and ensure a successful transition to home. To address this knowledge gap, this systematic review aims to examine modifiable and non-modifiable risk factors associated with readmissions and mortality among infants, children, and adolescents who are on LTMV-T.

## Methods

The protocol of this systematic review has been registered in the International Prospective Register of Systematic Reviews (PROSPERO) (ID CRD42024492773). The preparation of this review was guided by the Preferred Reporting Items for Systematic Review and Meta-Analysis (PRISMA) guidelines and checklist [26, 27].

### Data sources and search strategies

Five databases, PubMed, CINAHL, Web of Science, Embase, and Epistemonikos, were searched for studies. A systematic search strategy with medical subject headings (MeSH) and keywords related to long-term mechanical ventilation was developed with a medical librarian (LC) to identify studies. Hand searching and citation chaining were used to identify additional studies.

Studies examining risk factors associated with readmission and/or mortality in children less than 21 years of age who were discharged on LTMV-T were included in the systematic review. Descriptive studies with readmission and/or mortality and no association with risk factors specific to LTMV-T populations were also included. The age range (birth to 21 years) used in this review is based on guidance from the American Academy of Pediatrics, which recommends extending pediatric care into young adulthood for those with special health care needs [28]. For this review, LTMV-T is defined by the American Thoracic Society (ATS) and Thoracic Society of Australia and New Zealand/Australian Sleep Association as infants, children, or adolescents who failed to wean from ventilator support 3 months after ventilator initiation and are medically stable at home with invasive ventilation via tracheostomy for all or part of a 24 h day [29, 30]. Qualitative studies, case studies, systematic reviews, grey literature, and meeting abstracts as well as studies that only had in-hospital mortality or elective/planned readmission were excluded. Studies that did not report outcomes specific to LTMV-T populations, and from which LTMV-T-specific data could not be extracted or obtained from authors upon request, were excluded. Search strategies are provided in Additional File Table 1.

### Data management, study selection, and data extraction

Articles identified through search strategies were downloaded and managed on Zotero, a reference software program. After duplicates of studies were removed through Zotero, the remaining articles were imported in Covidence, where additional duplicates were removed. Two reviewers (LS, KM) independently screened titles/abstracts and full-text based on the eligibility criteria. Percent agreement and Cohen’s Kappa were 97% and 0.66 during title/abstract screening and 90% and 0.73 during full-text screening respectively. Two reviewers (LS, KM) independently used the Joanna Briggs Institute (JBI) cohort and cross-sectional checklist to assess the quality and risk of bias of included studies. Disagreement regarding study eligibility and quality between the two reviewers was resolved through discussion and a third reviewer (YF).

Data were extracted in Covidence using standardized Covidence extraction forms customized for this review. Data extraction forms in Covidence were piloted for each study design type before final data extraction. One reviewer (LS) extracted data from the eligible studies, and a second reviewer (KM) checked the accuracy and consistency of a randomly selected 5% of the extracted data. Disagreements during data extraction were resolved through discussion and a third reviewer (YF). Data items extracted where applicable included: 1) study characteristics: first author, publication year, country, methodology (e.g., study design, eligibility criteria, length of follow-up), setting (e.g., rural/urban, hospital, emergency department), primary aims, results, limitations, theoretical frameworks used, and funding sources; 2) Participant characteristics: demographics (e.g., gender, race, age), sample size, type and length of ventilation, and diagnosis; 3) Exposure: modifiable and non-modifiable risk factors; 4) Outcome: readmission (e.g., time to readmission, reasons for readmission, readmission rate), mortality (e.g., mortality rates, time to mortality, cause of death), and definitions/descriptions of study outcomes. For studies with cohorts mixed with LTMV-T and tracheostomy-only (no ventilator) populations as well as LTMV-T and non-invasive ventilation (NIV) populations, full text and supplemental materials were searched to extract subgroup data specific to children on LTMV-T. If subgroup data for risk of readmission or mortality were not reported, study authors were contacted for disaggregated data on readmission and mortality.

## Results

### Study characteristics

**Fig. 1:**
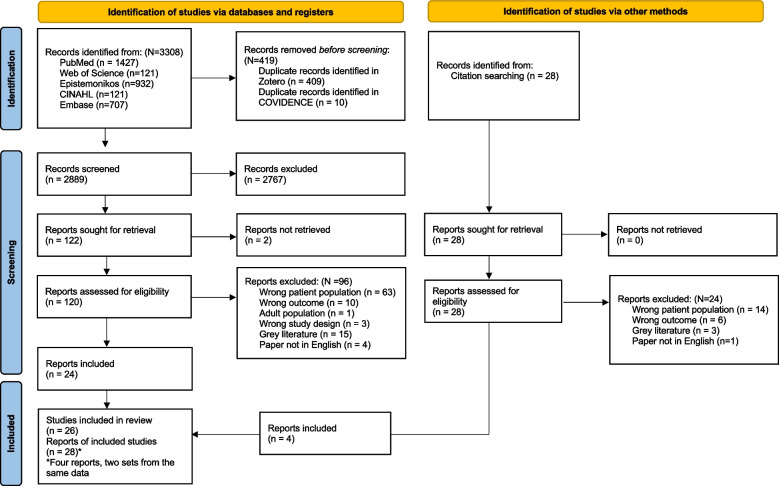
PRISMA 2020 flow diagram, Preferred reporting item for systematic review and meta analysis

Initial searches of the five databases identified 2,889 articles after duplicates were removed (Fig. 1). During title and abstract screening, 2,767 of the 2,889 articles were excluded due to irrelevance, leaving 122 articles for full-text review. There are 26 studies and 28 reports in this review because two reports used the same data from Children’s Hospital Los Angeles [31, 32] and two other reports used the same data from Riley Hospital for Children [33, 34].

Due to the common practice of grouping LTMV-T populations with tracheostomy-only and NIV populations in studies, three distinct subpopulations emerged when extracting data for children with LTMV-T. Group 1 studies included tracheostomy only (no ventilator) and/or LTMV-T populations (13/26 studies, 50%) [5, 31, 32, 35–44]. Group 2 focused on LTMV-T populations exclusively, our population of interest (9/26 studies, 35%). Group 3 included NIV and LTMV-T populations (4/26 studies, 15%) [45–48]. Table 1 shows the study and sample characteristics of the 26 studies that met the eligibility criteria, organized by these groupings.

**Table 1:** Study and Sample Characteristics

#	First author/year published/Country	Primary aim	Study design/Study period	Setting	Analysis	Child characteristics						
						Sample size, *n*/Ventilator type sample size n(%)	Age, median (IQR), mean(SD), n(%)	Female gender n(%)	Race/Ethnicity n(%)		Insurance n(%)	Underlying diagnosis (airway and/or respiratory, cardiac, neurologic/muscular, other)
**Group 1: Tracheostomy only (TO) *and* long-term mechanical ventilation via tracheostomy (LTMV-T) population**												
1	Lodge/2024/United States	Identify the association between home health nursing and 30-day same-hospital all-cause readmission and respiratory-related readmission among children < 21 years of age discharged with a new tracheostomy	Prospective cohort 2016–2023	Children’s Hospital Los Angeles	Multivariate logistic regression	*130*	1.12(0.7–4.94) median years	61(47)		19(14.6) Non-Hispanic White 15(11.5) Non-Hispanic Black 67(51.5) Hispanic 9(7) Asian or Pacific Islander 20(15.4) Other/Missing	89(68.5) Public 41(31.5) Private	41.5% Airways and/or respiratory 32.3% Cardiac 58.5% Neurologic/muscular
						86(66.2) LTMV-T						
						44(33.8) TO						
2	Beams/2023/United States	Determine risk factors and rates of frequent emergency department visits and hospitalizations within 2 years from tracheotomy among children < 18 years	Retrospective cohort 2015–2019	Children's Medical Center Dallas’s Children's Health Airway Management Program	Multiple logistic regression	*239*	0.6(0.3–3.9) median years at tracheostomy	119(50)		67(28) White 82(34) Black 65(27) Hispanic 8(3.3) Asian 16(6.7) Other	190(80) Medicaid 49(20) Private	95.4% Airway and/or respiratory 4.6% Other
						191(80) LTMV-T						
						48(20) TO						
3	Kukora/2023/United States	Identify factors associated with mortality in infants with neonatal conditions who underwent a tracheostomy in the first year of life	Retrospective cohort 2006–2017	Large Midwestern academic children’s hospital	Multivariate logistic regression	*224*	36(28–39) median weeks gestational age	94(42)		165(74) White 52(23) Black 7(3) Hispanic	NR	100% Airway and/or respiratory
						127(57) LTMV-T						
						97(43) TO						
4	Liu/2023/United States	Estimate risk factors and the 1-, 5-, and 10-year survival and decannulation rates of children (< 18 years) with a tracheostomy	Retrospective cohort 2009–2020	Children's Medical Center Dallas	Multivariable Cox proportional hazard regression	*551*	0.6(0.3–4.1) median years at tracheostomy	243(44)		308(66) White 154(33) Black or African American	267(67) Medicaid	NR
						358(69) LTMV-T						
						NR TO						
5	Van Horn^a^/2023/United States	Determine the association between rurality or public insurance status and 30-day readmission after tracheostomy in children ≤ 18 years	Retrospective cohort 2013–2017	49 PHIS hospitals	Multivariate logistic regression	*6746*	Nonrural: 4(0–56) median months Rural: 5(0–68) median months	2917(43)		3559 White 1521 Black 182 Asian 28 Pacific Islander 49 American Indian 951 Other	4329(64) Public 2193(32) Private 224(4) Self-pay/other/unknown	NR
						3291(49) LTMV-T						
						3455(51) TO						
6	Perez^a^/2022/United States	Determine rate of and factors associated with in-hospital mortality and 30-day readmission among children ≥ 31 days to < 21 years old with pulmonary hypertension who underwent tracheostomy	Retrospective cohort 2009–2017	37 PHIS hospitals	Multivariable mixed effects logistic regression	*793*	154(19.4) 31 days to < 3 months 355(44.8) 3 to < 6 months 131(16.5) 6 to < 9 months 46(5.8) 9 to < 12 months 107(13.5) 1 to < 21 years	359(45)		NR	522(65.8) Public 244(30.8) Commercial 23(2.9) Other 4(0.5) Unknown	100% Airway and/or respiratory and Cardiac
						390(65.7) LTMV-T						
						NR TO						
7	Phuaksaman/2022/Thailand	Evaluate the prevalence and factors associated with long-term outcomes of children ≤ 15 years old with tracheostomies	Retrospective cohort 2012–2020	King Chulalongkorn Memorial Hospital Pediatric critical care unit	Multivariate logistic regression	*85*	6.1(2.6–24) median months at tracheotomy	40(47)		NR	NR	67% Airway and/or respiratory 41.1% Neurologic/muscular 24.7% Other
						21(25) LTMV-T						
						64(75) TO						
8	Temur/2021/Turkey	Describe the outcomes of children who underwent congenital heart surgery and were discharged with home mechanical ventilation via tracheostomy	Retrospective cohort 2014–2018	Academic hospital	Kaplan–Meier survival analysis and log-rank test	*45*	6.4(12 days-6.5 years) median months	18(40)		NR	NR	100% Cardiac
						32(71) LTMV-T						
						NR TO						
9	Muesing/2020/United States	Identify the decannulation rates, all-cause mortality rates, and annual prevalence of tracheostomized children ≤ 16 years old over an 11-year span in Minnesota	Retrospective cohort 2008–2018	Minnesota pediatric home care company	Ordinary least squares regression	*175.9*^*b*^	NR(37.6) 0–2 years NR(31.7) 3–8 years NR(30.6) 9–16 years	NR(44)		NR(64.3) White NR(18.8) African American NR(4.5) Asian NR(3.0) American Indian or Alaskan Native NR(13) Other or Unknown	NR(53.5) Medicaid NR Other	41.2% Airway and/or respiratory 8.4% Cardiac 27% Neurologic/muscular 23.5% Other
						123.3^b^ LTMV-T						
						52.7^b^ TO						
10	Russell^a^/2018/United States	Identify risk factors associated with first hospital readmission due to a bacterial respiratory tract infection after tracheotomy among children ≤ 17 years old	Retrospective cohort 2007–2013	48 PHIS hospitals	Multivariable Cox Proportional hazard regression	*8009*	5(1–50) median months at admission	3391(42)		3873(48.4) Non-Hispanic White 1703(21.3) Non-Hispanic Black 1388(17.3) Hispanic 1045(13) Other	4940(61.7) Public 2482(31) Private 587(7.3) Other	86% Airway and/or respiratory 47% Neurologic/muscular 23.4% Other
						1826(23) LTMV-T						
						6183(77) TO						
11	Akangire/2017/Canada	Identify factors associated with readmission among ventilator-dependent infants within their first 2 years of life after tracheotomy	Retrospective cohort 2008–2015	Children's Mercy Kansas City Hospital's NICU	Logistic regression	*100*	43(43) 23–28 weeks 15(15) 28–34 weeks 15(15) 35–38 weeks 27(27) 38–42 weeks gestational age	49(49)		NR	NR	63% Airway and/or respiratory 9% Cardiac 4% Neurologic/muscular 24% Other
						54(54) LTMV-T						
						46(46) TO						
12	Russell^c^/2017/United States	Identify risk factors for readmission due to a bacterial respiratory tract infection within 12 months of discharge after tracheotomy among children 0–18 years old	Retrospective cohort 2005–2013	Children’s Hospital of Los Angeles	Multivariate logistic regression	*240*	5(1–37) median months at index hospitalization	96(40)		78(32.5) Non-Hispanic 162(67.5) Hispanic	213(88.8) Public 27(11.3) Other	95.9% Airway and/or respiratory 53.8% Neurologic/muscular 38.7% Other
						112(47) LTMV-T						
						128(53) TO						
13	Yu^c^/2017/United States	Determine readmission rates and identify factors associated with 30-day all-cause hospital readmissions among children ≤ 18 years undergoing tracheotomy	Retrospective cohort 2005–2013	Children’s Hospital of Los Angeles	Multivariate logistic regression	*273*	6(1–51) median months	107(39)		24(8.8) Non-Hispanic White 29(10.6) Non-Hispanic Black 181(66.3) Hispanic 39(14.3) Other/missing	241(88) Public 32(12) Private	91.2% Airway and/or respiratory 54.2% Neurologic/muscular 39.9% Other
						128(47) LTMV-T						
						145(53) TO						
14	Ortmann/2017/United States	Describe risk factors for mortality both in-hospital and after discharge in children < 18 years with congenital heart disease and tracheostomy	Retrospective cohort 2002–2015	Children's Mercy Hospital's PICU	Kaplan–Meier survival analysis and log-rank test	*33*	37.6(range 33–40) mean gestational age	23(50)		NR	NR	100% Cardiac
						21(64) LTMV-T						
						12(36) TO						
**Group 2: LTMV-T**												
15	Akangire/2022/United States	Identify the survival rate, ventilator liberation, and factors for decannulation in infants with severe BPD who had a tracheostomy in their first year of life	Retrospective cohort 2004–2017	Children's Mercy Kansas City level IV NICU and Tracheostomy and Home Ventilator clinic	Descriptive	*98*	4(3–5) median months at tracheostomy	47(48)	56(57.1) White 29(29.6) Black 3(3.1) Hispanic 1(1.0) Asian 3(3.1) Multiracial 1(1.0) American Indian 3(3.1) Native Hawaiian or Pacific Islander 2(2.0) Other		66(68) Public 11(11.3) Private 18(18.6) Combination 2(2.1) Self-pay	100% Airway and/or respiratory
16	Giambra ^a^/2021/United States	Identify regional variations in characteristics, costs, length of stay, and readmissions among children with established LTMV-T	Retrospective cohort 2014	49 PHIS hospitals	Mixed Poisson regression	*2233*	644(28.8) < 2 years 597(26.7) 2 to < 6 years 443(19.8) 6 to < 12 years 360(16.1) 12 to < 18 years 189(8.5) ≥ 18 years	975(44)	Race: 1355(62.8) White 399(18.5) Black 403(18.7) Other		1540(69.2) Public 644(28.9) Commercial 43(1.9) Self-pay/no charge/other	NR
									Ethnicity: 477(22.7) Hispanic or Latino 1620(77.3) Not Hispanic or Latino			
17	Borges/2020/Brazil	Describe patient characteristics and predictors of hospital readmission and death among children LTMV-T over 10 years	Cross-sectional retrospective 2007–2016	Brazilian Home Care Services	Binary logistic regression	*27*	4.04(3.96) mean years at home admission	11(41)	NR		NR	7.4% Airway and/or respiratory 59.2% Neurologic/muscular 33.3% Other
18	Rogerson/2020/United States	Describe the characteristics and costs of care among children < 18 years of age on LTMV-T within the first year after discharge from the intensive care unit	Retrospective cohort 2015- 2017	Riley Hospital for Children	Descriptive	*50*	4.5(NR) median months at tracheotomy	17(34)	26(52) White 16(32) Black 4(8) Hispanic 4(8) Other		NR	68% Airway and/or respiratory 4% Cardiac 28% Neurologic/muscular
19	Ertugrul/2017/Turkey	Describe clinical characteristics and follow up results of children on LTMV-T after discharge	Retrospective cohort NR	Hacettepe University Ihsan Dogramaci children’s hospital's PICU	Descriptive	*61*	8.5(range 2–196) median months	NR	NR		NR	4.9% Airway and/or respiratory 6.6% Cardiac 85.2% Neurologic/muscular 3.3% Other
20	Henningfeld/2016/United States	Describe the experience of children on LTMV-T who successfully decannulated	Retrospective cohort 1999–2011	Children’s Hospital of Wisconsin’s tracheostomy-home mechanical ventilation clinic	Descriptive	*46*	6.0(NR) median months at ventilator initiation	21(46)	21(46) Caucasian 18(39) Black 4(9) Hispanic 2(4) Asian 1(2) American Indian		High-risk zip code: 18(39) Public 0 Private Low-risk zip code: 16(35) Public 12(26) Private	98% Airway and/or respiratory 2% Neurologic/muscular
21	Cristea^d^/2015/United States	Determine the association between median household income and mortality among children with BPD discharged on LTMV-T	Retrospective cohort 1984–2010	Riley Hospital for Children Pediatric Pulmonology Clinic Home Ventilator Program	Kaplan–Meier survival analysis and log-rank test	*94*	26(25–27) median gestational age	39(41)	65(69.1) White 22(23.4) Black 7(7.5) Other: Hispanic, Asian		57(60.6) Medicaid 20(21.3) Private 17(18.1) Private/Medicaid	100% Airway and/or respiratory
22	Cristea^d^/2013/United States	Determine the incidence, survival, liberation, and decannulation of children with severe BPD discharged on LTMV-T	Retrospective cohort 1984–2010	Riley Hospital for Children Pediatric Pulmonology Clinic Home Ventilator Program	Cox proportional hazard	*102*	26(25–27) median gestational age	38(37)	70(68.6) White 24(23.5) African American 8(7.8) Other: Hispanic/Asian		61(63) Medicaid 20(21) Private 16(16) Private/Medicaid	100% Airway and/or respiratory
23	Kun/2012/United States	Identify the 12-month incidence and risk factors of non-elective readmission among children < 21 years of age with chronic respiratory failure discharged to private home or sub-acute facility on LTMV-T	Retrospective cohort 2003–2009	Children’s Hospital Los Angeles	Generalized estimated equation multivariable regression	*109*	Readmitted: 0.83(0.58–2.1) median years at ventilator initiation Not readmitted: 1.1(0.66–9.7) median years at ventilator initiation	43(39)	13(12) White 18(16.5) African American 72(66) Hispanic 6(5.5) Asian/Pacific Islander/Middle Eastern		70(64) Public 39(36) Private	61% Airway and/or respiratory 39% Neurologic/muscular
24	Gilgoff/2003/United States	Describe the morbidity, mortality, and quality of life among discharged children who were initiated on LTMV-T before 6 years of age	Retrospective cohort 1980–2000	Rancho Los Amigos National Rehabilitation Center (RLANRC)	Kaplan–Meier survival analysis and log-rank test	*39*	2.08(range 0–5.08) mean years at ventilator initiation	11(28)	NR		NR	100% Neurologic/muscular
**Group 3: Non-invasive mechanical ventilation (NIV) and LTMV-T**												
25	Özcan/2021/Turkey	Determine the risk factors of first nonscheduled hospital admissions of children ≤ 18 years old on ventilation	Retrospective cohort 2014–2020	Ankara University	Univariate logistic regression model	*97*	23(10–91) median months	41(42)	NR		NR	17.5% Airway and/or respiratory 61.9% Neurologic/muscular 20.6% Other
						70(72) LTMV-T						
						27(28) NIV						
26	Pavone/2020/Italy	Describe the characteristics, ventilator liberation, and mortality rates of children ≤ 18 years old on long-term ventilation discharged to home or long-term facility	Retrospective cohort 2000–2017	Pediatric Hospital Bambino Gesù	Descriptive	*432*	2.1(0.8–7.8) median years at ventilator initiation	57(49)	NR		NR	16% Airway and/or respiratory 73% Neurologic/muscular 11% Other
						117(27) LTMV-T						
						315(73) NIV						
27	Amin/2014/Canada	Describe the trends and clinical outcomes of children on NIV or LTMV-T in a home ventilation program	Retrospective cohort 1991–2011	Home Ventilation Program at the Hospital for Sick Children	Descriptive	*379*	9.29(4.4–14.9) median years	27(41)	NR		NR	18.2% Airway and/or respiratory 78.8% Neurologic/muscular 3% Other
						66(17) LTMV-T						
						313(83) NIV						
28	Pekcan/2010/Turkey	Examine the characteristics and outcomes of children who were discharged with NIV or LTMV-T over a four-year period	Prospective cohort 2003–2007	Hacettepe University Pediatric Chest Diseases Unit	Descriptive	*27*	59.4(range 1 day-15 years) mean months	8(30)	NR		NR	22.2% Airway and/or respiratory 11.1% Cardiac 59.3% Neurologic/muscular 7.4% Other

*TO* tracheostomy only, *LTMV-T* long-term mechanical ventilation via tracheostomy *NR* not reported *PHIS* Pediatric Health Information System, *NICU* neonatal intensive care unit, *PICU* pediatric intensive care unit *BPD* bronchopulmonary dysplasia *NIV* non-invasive mechanical ventilation. See additional file Tables 4 and 5 for detailed readmission and mortality results

^a^Studies that used the same Pediatric Health Information System (PHIS) national database

^b^Average daily patient census in Minnesota

^c^Reports that use the same data/population from Children’s Hospital Los Angeles and therefore count as one study

^d^Reports that use the same data/population from Riley Hospital for Children and therefore count as one study

Of the 26 studies included, one was cross-sectional, two were prospective cohort studies, and 23 (88%) were retrospective cohort studies using electronic health records. Published between 2003 and 2024, these studies examined cohorts of children on LTMV-T as early as 1980 to as recent as 2023, over periods ranging from 1 to 27 years. Most studies (77%) were conducted in urban, single-center, pediatric hospitals, while two (8%) used home care services databases [5, 24] and four (15%) utilized the Pediatric Health Information System, a United States national database [17, 38, 39, 42]. Geographically, the studies covered six countries: Canada (2), Brazil (1), Italy (1), Thailand (1), Turkey (4), and the United States (17).

### Sample characteristics

Study sample sizes ranged from 27–8,009 infants or children, with most studies focusing on patients ≤ 18 years (8/26, 31%). Children on LTMV-T were young with a median age of 4.5 months to 8.5 months and had an underlying diagnosis that was predominately airway and/or respiratory (50%) [15, 33, 34, 49, 49, 50]. The majority of participants had public health insurance (e.g., Medicaid), ranging from 54 to 88% [5, 15, 17, 31, 34, 35, 37–39, 42, 51–53], and 56–100% of children were discharged home following tracheostomy and/or ventilator initiation [1, 15, 17, 24, 34, 38–40, 42, 43, 49, 51, 52, 54].

### Risk of bias and quality assessment

**Fig. 2:**
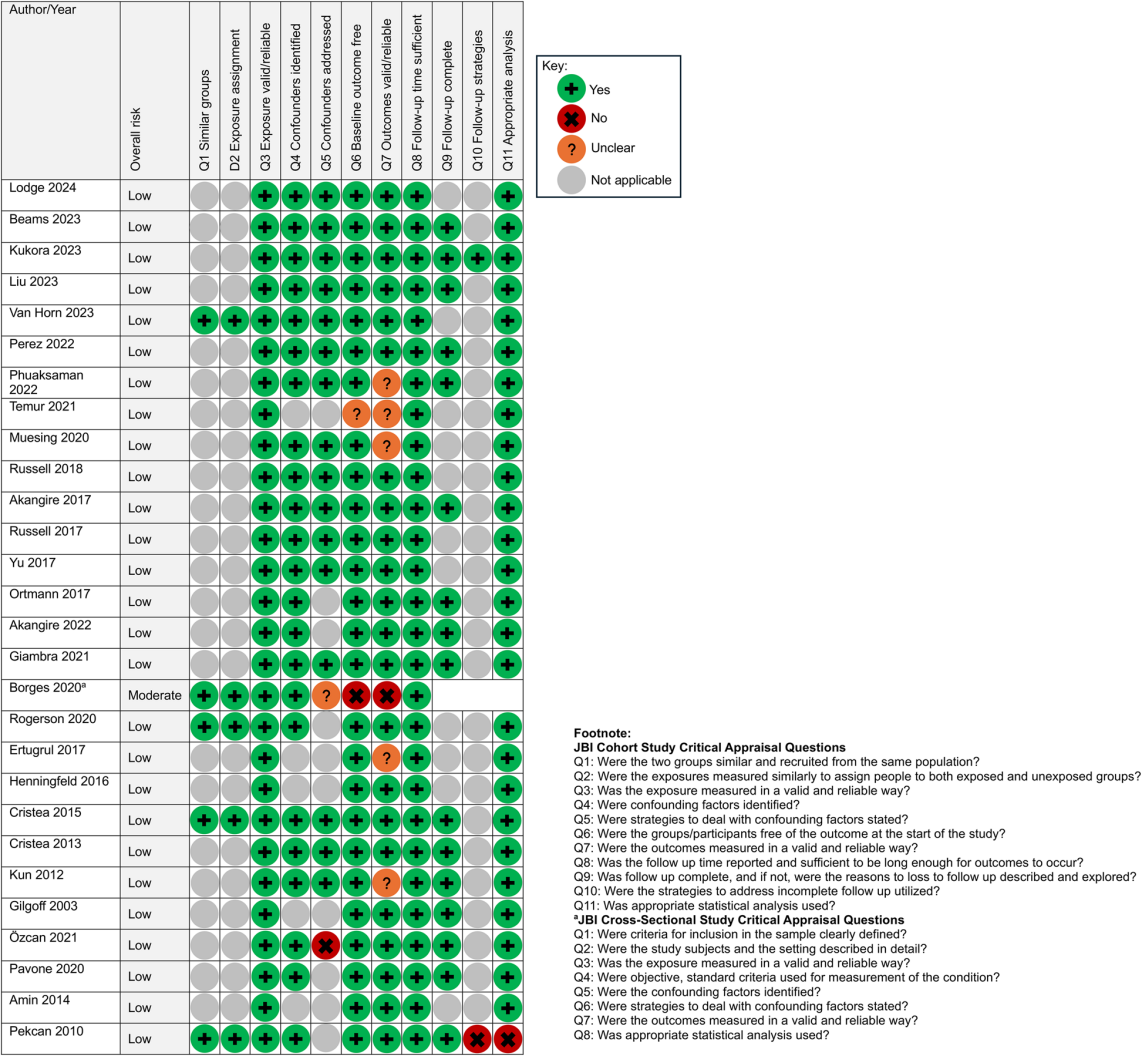
Risk of bias assessment: the Joanna Briggs Institute (JBI) critical appraisal checklist

The included studies were assessed to have a low to moderate risk of bias, primarily attributable to their study designs (Fig. 2). Of the 26 studies, 12 (46%) did not control for or address potential confounding factors in either the study design or the analysis. Ten of these studies were descriptive and only conducted univariate or bivariate analyses [41, 44, 46–50, 52, 54, 55]. Only five studies included a non-exposed cohort for comparison. Outcome measurement methods were unclear in six studies due to limited detail on electronic health record data extraction.

## Readmission and mortality outcomes

### Readmission

Readmission outcomes were examined in 19 studies (Additional File Tables 2 and 4), with most focusing on children from tracheostomy-only and/or LTMV-T populations (47%) or LTMV-T-only populations (53%). Reporting of readmission outcomes varied significantly, including metrics such as mean or median readmissions per year, number of emergency visits, and total readmissions during specified periods. Many studies reported readmissions 30-days post-discharge, but time frames also included one to two years post-discharge, first two to four years of life, specified study periods, and time to the first bacterial respiratory tract infection readmission.

**Fig. 3:**
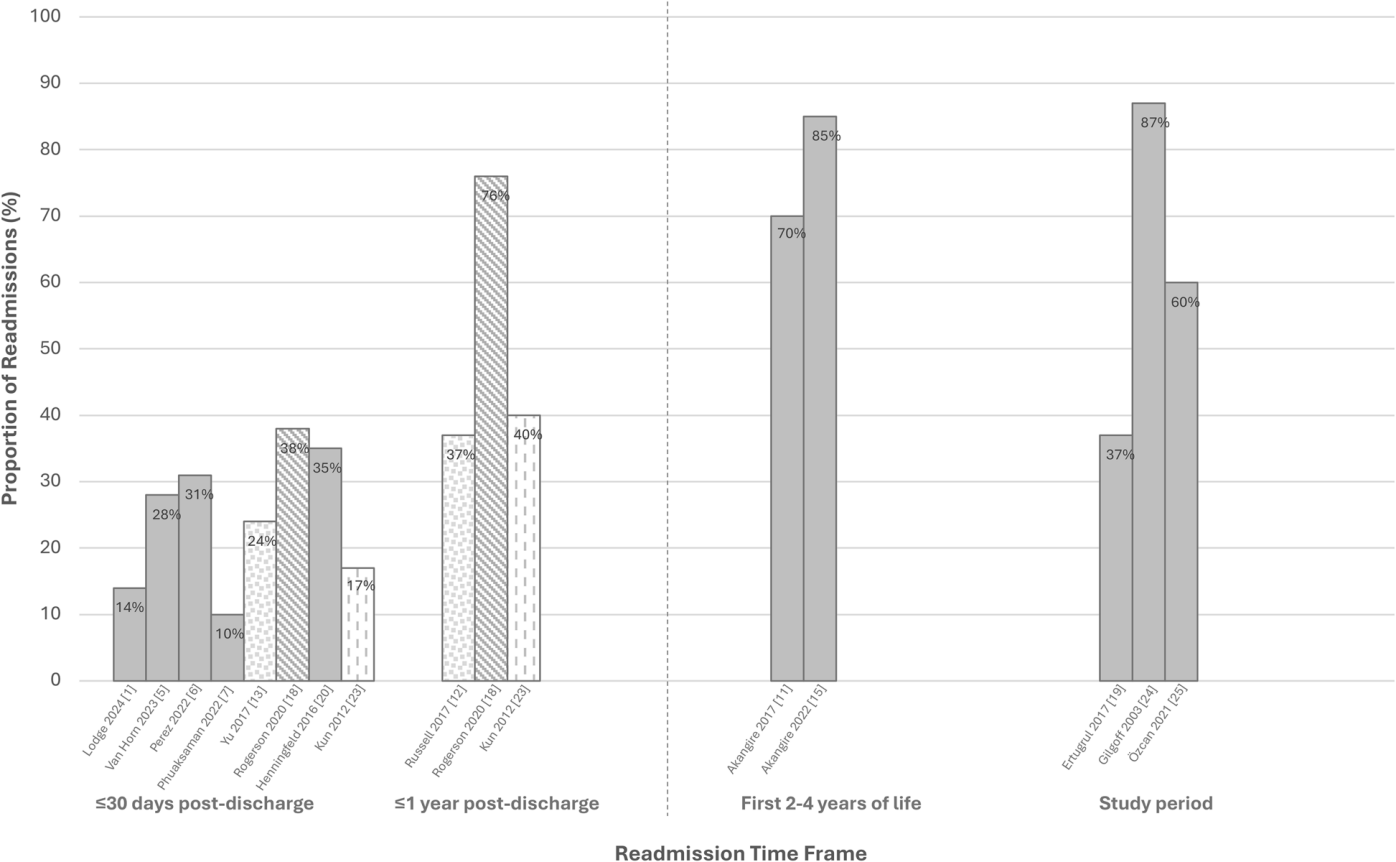
Proportion of readmissions among children on long-term mechanical ventilation via tracheostomy (*n* = 13). This figure illustrates 14 articles (13 studies) that report the proportion of children on long-term mechanical ventilation via tracheostomy who experienced a readmission within 30 days and 1 year post-discharge, as well as a readmission within the first 2 to 4 years of life or during a study period. Study periods ranged from 6 to 10 years. Each bar represents one study with the author and year specified below. Two articles reported readmission at 2 different time points (within 30 days and 12 months from discharge). The stripped pattern bars are reflective of the same articles. The dotted pattern bars are indicative of different articles that used the same patient population. Children who only had a tracheostomy and no ventilator as well as children on non-invasive ventilation were not included in this figure

Figure 3 illustrates the frequent occurrence of readmission across time frames, specifically among children on LTMV-T. Approximately one-third of children experienced a readmission within the first-year post-discharge. Median overall readmissions per child were approximately 2 in the first-year post-discharge [17, 49], with half occurring in the first three months in several studies [15, 49]. In contrast, the median time to first bacterial respiratory infection readmission was reported as 275 days [42]. Readmission rates were higher over longer study periods (37–87%) and during the first two to four years of life (70–85%); however, most readmissions still occurred early, within the first month or year post-discharge [43, 45, 52, 54, 55]. Readmissions were predominantly respiratory-related, accounting for 30–75% across all time frames, with tracheitis (17–48%) and tracheostomy-related complications (6–31%) such as decannulation and obstruction contributing to a significant amount within the first-year post-discharge [15, 24, 31–33, 35, 38–40, 43, 45, 54].

### Factors associated with readmission

**Fig. 4:**
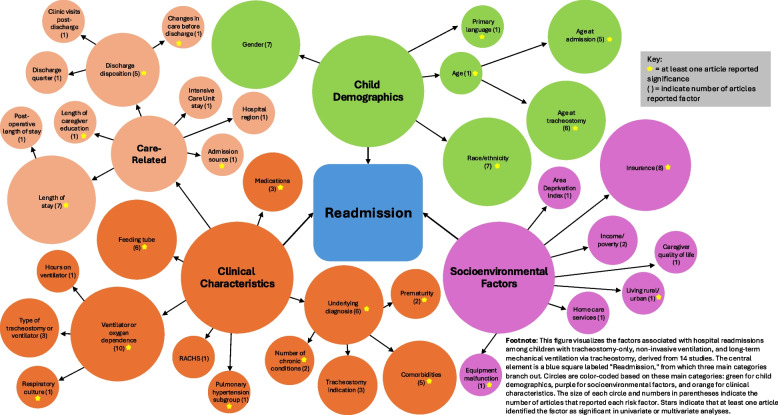
Readmission factors across all pediatric cohorts (*n* = 14)

Of the 14 studies examining risk factors associated with readmission, the majority (57%) focused on tracheostomy only and/or LTMV-T populations [31, 32, 35, 38–40, 42, 43, 53], while 36% focused exclusively on LTMV-T populations [15, 17, 34, 49, 55]. Figure 4 highlights readmission factors across all populations (NIV, LTMV-T, tracheostomy-only), with the most examined factors including child demographics (age, race, gender) and clinical characteristics such as comorbidities, underlying diagnosis, discharge disposition, ventilator dependence, length of stay, and feeding tube use. Of socioenvironmental factors, insurance status was the most common factor included in statistical models [15, 17, 31, 35, 38, 42, 53]. Only two studies examined economic stability and one study provided the most comprehensive assessment by utilizing the Area Deprivation Index [34, 35].

Associations between risk factors and readmissions were poorly reported in studies focusing on LTMV-T specific populations, with only 3 studies adjusting for possible confounding variables. Age, insurance, admission source (i.e., clinic, hospital transfer), discharge disposition (i.e., home, skilled facility), and number of complex chronic conditions other than respiratory were associated with number of readmissions; however incidence rate ratios were not reported [17]. Within the same study, regional (i.e., Midwest), gender, and racial differences were not found to be significant. Gastrostomy tube placement (aOR = 4.41, 95% CI: 1.1–18) at the index hospitalization increased risk of 30-day readmission, while neuromuscular comorbidities, public health insurance, and home health nursing were not significant [53]. In contrast, one study found no statistical association between 12-month readmission and any clinical characteristic (e.g., age, neurodevelopmental delay, epilepsy, feeding tube, ventilator type, and discharge disposition) [56]. Another study identified lower median hospitalization rates/year with a respiratory diagnosis among children with high Zip code Annual Household Income (Z-AHI) compared to low Z-AHI, 0.55 and 0.92 respectively, although findings were not statistically different [34].

In contrast, more data was available among cohorts mixed with tracheostomy only (no ventilator) and LTMV-T populations. Three studies identified Black race (OR = 2.01, 95% CI 1.2–3.7) and Hispanic ethnicity (aOR = 2.0, 95% CI: 1.1–3.9) as increased risk for readmissions [32, 35, 42]. Conversely, race and ethnicity were not significant factors among LTMV-T specific studies [15, 17]. Children who were younger at admission [39, 42], had multiple complex chronic conditions [17, 42], and reported their primary language as Spanish (OR: 3.86, 95% CI: 1.5–10.1) [35] had an increased risk for readmission. Public insurance (OR = 1.24, 95% CI: 1.1–1.4) compared to private also increased risk for readmission [38, 42]. While two studies reported increased risk of readmission with feeding tube devices (aOR = 4.41, 95% CI: 1.1–18) [42, 53], one study identified a decreased risk of 30-day readmission (aOR = 0.42) [31]. Discharge to home, compared to post-acute care settings, increased the risk of respiratory-related readmission (aHR 1.19, 95%CI: 1.1–1.3) [42]. Among LTMV-T specific populations however, discharge with home health services compared to no services was not significant [53]. Five studies identified ventilator dependence as a significant risk factor for readmission with an odds ratio ranging from 2.7–29 [31, 35, 43, 45]. Only one study reported decreased risk with ventilator dependence for respiratory-related readmissions [42].

### Mortality outcomes

**Fig. 5:**
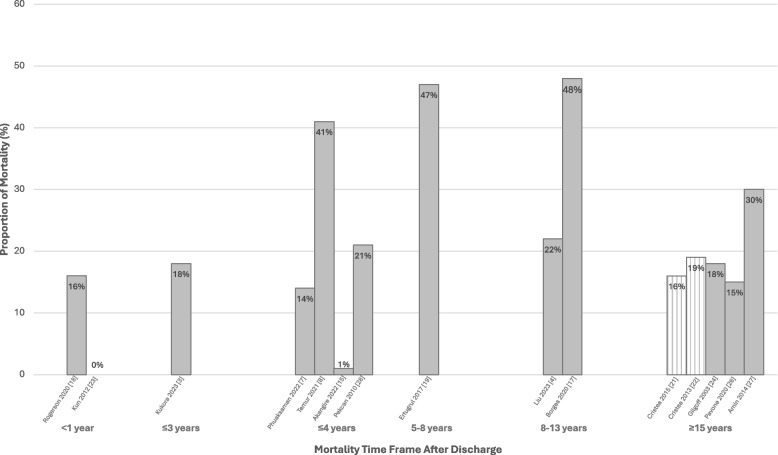
Proportion of mortalities after discharge among children on long-term mechanical ventilation via tracheostomy (*n* = 14). This figure illustrates 15 articles (14 studies) that report the proportion of children on long-term mechanical ventilation via tracheostomy who died within 1 year, 3 years, 4 years, 5-8 years, 8-13 years, or greater than 15 years from discharge. Each bar represents one study with the author and year specified below. The stripped pattern bars are reflective of different articles that used the same patient population. Temur 2021 had a cardiac specific population and Borges 2020 had children who were all on palliative care. Children who only have a tracheostomy and no ventilator as well as children on non-invasive ventilation are not included in this figure

Mortality outcomes were examined across 17 studies, spanning 1 to 27 years of follow-up, and included the number of deaths, survival rates, median age and time to death, causes, and locations of death (Additional File Tables 2 and 5). Among the 17 studies, 7 (41%) focused on tracheostomy only and/or LTMV-T populations, 7 (41%) on LTMV-T populations, and 3 (18%) on NIV and LTMV-T populations. Median age at death ranged from 1.42 to 5.03 years [34, 36, 47, 52]. Deaths often occurred within the initial two years post-discharge, with an average and median time to death from 7.5–8.5 months and 1.6 years respectively [36, 40, 48]. Despite variability in the location of death across the studies, a higher proportion of deaths (23% to 64%) occurred at home [36, 41, 44, 47, 48, 52, 54]. Figure 5 highlights the variability in the proportion of deaths post-discharge and considerable risk within the first two years, specifically among children on LTMV-T. Within the first year, mortality ranged from 0 to 16%, increasing to 18%–41% over 3 to 4 years [36, 41, 48, 52]. Longer follow-ups reported higher mortality rates; however, majority of deaths occurred closer to the date of discharge [36, 41, 44, 48, 49, 52]. Tracheostomy-related complications accounted for nearly a quarter of deaths across all time periods [44, 49, 52, 54, 55].

### Factors associated with mortality

**Fig. 6:**
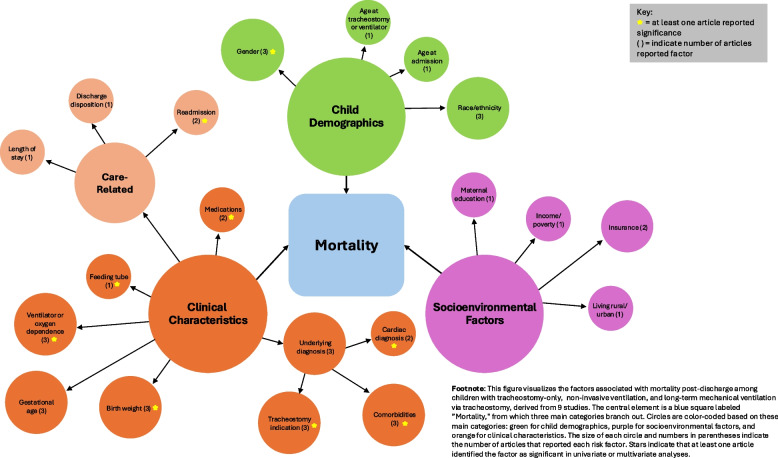
Mortality factors across all pediatric cohorts (*n* = 9)

Risk factors for mortality varied widely across 9 studies and are illustrated in Fig. 6. Three studies (33%) were among tracheostomy and/or LTMV-T [36, 37, 44], 5 (56%) LTMV-T [24, 33, 34, 49, 54, 55], and 1 (11%) NIV and LTMV-T [48]. The most examined factors included child demographics (gender, race, ethnicity) and clinical characteristics (gestational age, birth weight, indication for tracheostomy, comorbidities, underlying diagnosis, ventilator dependence). Socioenvironmental factors were only assessed in several studies including insurance type [33, 37], income [34], maternal education [48], and distance from hospital [48].

Only 3 studies adjusted for possible confounding variables. Among tracheostomy only and/or LTMV-T populations, an airway obstruction diagnosis (aOR = 1.53, 95% CI: 1.0–2.3), tube feeding device (aOR = 1.14, 95% CI: 1.0–1.3), severe neurologic disability (HR = 2.79, 95% CI: 1.6–4.8), congenital heart disease (HR = 1.69, 95% CI: 1.1–2.7), and ventilator use at discharge (HR = 2.04, 95% CI: 1.1–3.8) increased risk of mortality [36, 37]. The only LTMV-T specific study that controlled for confounding identified birthweight of ≤ 750 g, compared to > 750 g, was associated with an increased risk of mortality (HR = 0.34, 95% CI: 0.1–1.0) [33]. No child demographics or socioenvironmental factors were statistically significant among children on LTMV-T.

Among univariate analyses of children on LTMV-T, those who received physiotherapy (i.e., cough assist, percussions, vibrations) had lower mortality rates compared to children who did not receive physiotherapy (51% vs. 80%, *p* < 0.01) [55]. In an unadjusted binary logistic regression model, children who were readmitted within ≤ 6 months of discharge had a 10% greater chance of death [24]. Although not statistically significant (*p* = 0.21), mortality rates were higher among those with a neurologic disorder (29%) compared to respiratory disorder (12%) during the first 12 months from discharge [49]. One study identified economic differences in mortality, reporting that children on LTMV-T with lower Z-AHI had a higher mortality rate (24%) compared to those with higher Z-AHI (3%, *p* = 0.003) [34]. There were no clear patterns of significant risk factors across mortality time frames. See Additional File Table 3 for a detailed report of risk factors and significance.

## Discussion

### Major findings

With a growing pediatric LTMV-T population, understanding risk factors for poor outcomes post-discharge and providing means to successfully care for the child in the home setting is important. This systematic review is the first to our knowledge, to provide a comprehensive summary of readmission and mortality outcomes and to identify factors associated with readmission and mortality among children on LTMV-T. Among the 26 included studies, only 9 focused specifically on children with LTMV-T and 5 examined factors for readmission and mortality among this patient population. The limited number of studies generated from our review emphasizes the paucity of literature on children dependent on LTMV-T.

Major findings from our review demonstrated that readmissions are common occurrences among children on LTMV-T. For most studies, at least 25% of children on LTMV-T experienced readmission regardless of the readmission time frame [31, 32, 36, 38–40, 43, 49, 50, 52, 57]. Close follow-up and interventions for these children should focus on the first two years from discharge because the majority of studies reported that at least 50% of readmissions occurred within a timepoint in the first two years from discharge [24, 35, 43, 45, 52]. High readmission rates, especially within the first two years of discharge, indicate potential challenges during the discharge process and reflect difficulties experienced by caregivers when transitioning to home. During the transition from hospital to home, caregivers of children on LTMV-T are often overwhelmed, exhausted, anxious, and unprepared for discharge [58, 59]. Caregivers who report higher strain and lower mental health-related QOL are more likely to have lower confidence in avoiding future hospitalizations for the child [60]. In addition, gaps in care during discharge, including inadequate teaching, gaps in services and limited community resources, and poor communication between hospital and community providers, reduce caregiver confidence in providing interventions and may increase the risk of poor health outcomes [61–64].

Our review of mortality outcomes also highlighted the importance of the initial post-discharge period. The majority of deaths occurred closer to the time of discharge, regardless of follow-up duration, and mortality was as high as 16% within the first year [36, 41, 48, 49]. The primary causes of both readmission and mortality were predominately respiratory-related with about a quarter of cases being preventable complications such as accidental tracheostomy dislodgement/obstruction. While previous studies have suggested that underlying diseases are the primary cause of mortality, recent studies and this systematic review have demonstrated that unanticipated deaths are also a major cause [18, 56]. Attention should be directed towards modifiable factors to prevent mortality, such as the care quality provided at home or during follow-up and focused training on management of tracheostomy complications. Appropriate discharge training on infection prevention and tracheostomy care may prevent some respiratory-related readmissions and/or deaths. To mitigate such poor health outcomes, the ATS developed clinical practice guidelines for pediatric LTMV-T. The guidelines provide recommendations on standardized discharge criteria, training of caregivers, equipment for monitoring, and emergency preparedness [29]. However, despite publication in 2016, it is not clear how many pediatric hospitals utilize these guidelines in discharge preparation of families. In this systematic review, 13 studies had a discharge program for children on LTMV-T [24, 31, 33, 35–37, 40, 41, 49, 55, 57, 57]; however, only two studies indicated the utilization of ATS guidelines to inform discharge planning and training [43, 49, 52]. Variations in management and care practices for these children could have impacted the readmission and mortality outcomes observed; however, the absence of detailed data made such comparisons in the review not possible.

The observed readmission and mortality rates across cohorts in this review (1980–2024) displayed minimal variation, indicating limited progress in improving these outcomes over time. Current discharge programs are often one-size-fits-all and may fail to address the needs of high-risk groups. Identifying risk factors can help guide clinicians in recognizing high-risk groups, implementing tailored discharge programs, and ultimately reducing readmission and mortality rates. However, the insufficient examination and significance of the number of risk factors among children on LTMV-T in this review prevent drawing substantial conclusions. The majority of risk factors associated with readmission (e.g., age, admission source, disposition, complex chronic conditions, gastrostomy tube) and mortality (e.g., low birth weight, readmission within 6 months, receipt of respiratory physiotherapy) were clinical characteristics among LTMV-T [15, 17, 24, 33, 55]. Such clinical characteristics usually indicate level of acuity and are therefore expected to increase healthcare utilization or death; however, many of such characteristics are not modifiable for intervention development.

Our systematic review demonstrates the role of social determinants of health (SDOH) in differential outcomes for children with tracheostomies. According to the World Health Organization, SDOH, or the conditions in which individuals live, work, and play in account for as much as 80% to 90% of modifiable contributors to overall health outcomes [65]. The United States Department of Health and Human Services’ Healthy People 2030 goals have also emphasized a need to examine SDOH in the context of health and health care. SDOH have been identified as risk factors for outcomes such as hospital length of stay and costs among children with a tracheostomy only and medically complex care populations [66, 67]. Few studies in this review examined SDOH beyond health care access (i.e., insurance type) among children on LTMV-T [68]. However, even health care access can be further measured for children on LTMV-T such as transportation to medical appointments, access to clinicians for advice/urgent situations, and communication with medical equipment services. In this review, public insurance and self-reported Black race and Hispanic ethnicity, proxies of racial inequities, were identified as factors that increased risk of readmission among children with tracheostomy only and/or LTMV-T. This highlights the significance SDOH, and racial disparities may have on health outcomes. It also underscores the need to further examine structural inequities, such as access to home health nursing, among marginalized groups of children on LTMV-T. However, as demonstrated in this review, SDOH among children with LTMV-T are not described well in the literature and requires further research.

### Limitations of studies and the systematic review

Despite the valuable insights gained from this systematic review, several limitations should be acknowledged. Studies primarily used electronic health record reviews for data which limited the potential variables and prospective analysis. The heterogeneity in age groups, underlying diseases (e.g., bronchopulmonary dysplasia, congenital heart disease), time of follow-up, time frames for outcomes, outcome measures, and reporting styles posed challenges in synthesizing the evidence and made meta-analysis not possible. For example, some studies reported all-cause readmission, while others reported acute respiratory distress-related readmission or planned and unplanned readmissions. Most studies combined tracheostomy-only (no ventilator) and LTMV-T populations despite differences in acuity and ventilation as a significant risk factor. Notably, tracheostomy only populations had more granular data (e.g., cause of readmission/death, time to event, the median age at readmission/death) available compared to children on LTMV-T. Subgroup analyses were also not available to draw further conclusions regarding factors associated with readmission and mortality for children on LTMV-T. Further research should strive for standardized reporting to facilitate comparisons and meta-analyses (e.g., similar outcomes measures, start of follow-up). Time-to-event analyses should also be conducted to improve understanding of periods most vulnerable to poor health outcomes and the associated risk factors. Generalizability of study findings may be limited, and results of readmission and mortality rates may be skewed. Many studies were conducted in high-income countries and in urban, single-center pediatric hospitals specialized in caring for this patient population. Readmission and mortality rates may be higher in non-pediatric hospitals located in rural settings or low- and middle-income countries with limited resources.

This systematic review was restricted to the English language for included studies and may exclude key studies written in other languages or perspectives from low- and middle-income countries. In addition, included studies were limited to peer-reviewed journals which omitted relevant conference abstracts and grey literature. Due to the grouping of children on LTMV-T among other patient populations such as medically complex care, relevant articles may have been missed during title/abstract screening. A major strength of this systematic review is the use of PRISMA 2020 guidelines to decrease bias and increase the review's quality, transparency, and reproducibility [27]. A thorough investigation of available literature was achieved by utilizing five databases and search terms that increased the sensitivity of finding relevant studies. Furthermore, piloting of search strategies and citation chaining increased the likelihood of reflecting most studies on this topic and the robustness of this review. Multiple independent reviewers during the screening and data extraction process reduced potential bias.

### Implications

Findings from this review can inform future research on risk factors associated with readmission and mortality with the goal of identifying potential interventions. Larger, prospective cohort studies among children on LTMV-T are needed to power analyses of risk factors and gather more granular data on modifiable risk factors/SDOH. The limited reporting of caregiver characteristics within this review also underscores the need for future studies to explore the impact of caregiver-related factors on outcomes. Other variables of interest for future research include language and numeracy barriers or communication challenges during discharge. Considering the number of respiratory infection-related readmissions, infection risk factors such as respiratory microbiome, number of siblings at home, and home health should also be further explored. To compare severity within the population of LTMV-T, ventilator modality, hours on ventilator, and other granular population specific variables should be collected. Variations in practices across hospitals should be examined to understand the extent hospital management influences health outcomes. Even policies or cultural practices should be compared across studies. For example, study authors in this systematic review who published from Turkey and Thailand stated that home nursing was not an option for families and children on LTMV-T, and may also be the case in the United States in poor medical access areas. Such differences in healthcare norms, policies, and cultures should be accounted for in analyses.

Risk factors identified through these studies can inform clinicians of high-risk groups to provide individualized care during discharge planning and follow-up post-discharge. Moreover, discharge programs can be developed to anticipate the needs of specific individuals. Specialized transition support and coordination can be developed for targeted groups. Lastly, risk factors identified in this study can be used to identify resource deficiencies within the community and inform policies. Policies can be implemented to enhance the availability of home care nursing, improve caregiver compensation to alleviate the burden on caregivers, and boost community resources to support the well-being and development of both caregivers and children.

## Conclusion

This systematic review, to our knowledge, is the first comprehensive summary of readmission and mortality outcomes, along with associated risk factors for children on LTMV-T. The first two years post-discharge are a vulnerable period for children on LTMV-T due to high rates of readmission and mortality. At least a quarter of these events are due to preventable causes, such as tracheostomy complications. The findings from this review highlight the need to standardize outcome measures and reporting, explore socioenvironmental risk factors, and address readmission and mortality through intervention development to improve the care and outcomes of children on LTMV-T.

## Supplementary Information

Additional file 1: Table S1. Search Strategies; Table S2. Summary of Readmission and Mortality Outcome Measures; Table S3. Factors for Readmission and Mortality; Table S4. Summary of Readmission Measures and Results; Table S5. Summary of Mortality Measures and Results

## Data Availability

All data generated or analysed during this study are included in this published article and its supplementary information files.

## Abbreviations

LTMV-T: Long-term mechanical ventilation via tracheostomy
QOL: Quality of life
PROSPERO: Prospective Register of Systemic Reviews
PRISMA: Preferred Reporting Items for Systematic Review and Meta-Analysis
MeSH: Medical subject headings
ATS: American Thoracic Society
NIV: Non-invasive ventilation
JBI: Joanna Briggs Institute
Z-AHI: Zip code Annual Household Income
SDOH: Social determinants of health
