# Supplementary material for: Readmission and mortality among children requiring long-term mechanical ventilation via tracheostomy: a systematic review
Source: BMC Pulm Med. 2025 Aug 11;25:385. doi: 10.1186/s12890-025-03818-3 (PMC12341097; doi:10.1186/s12890-025-03818-3)
Supplement: Supplementary file 1 — Additional file 1: Table S1. Search Strategies; Table S2. Summary of Readmission and Mortality Outcome Measures; Table S3. Factors for Readmission and Mortality; Table S4. Summary of Readmission Measures and Results; Table S5. Summary of Mortality Measures and Results [file 12890_2025_3818_MOESM1_ESM.pdf]

Additional Files

Table 1. Search Strategies

Search	PubMed query
#1 Sample	infant [mh] OR baby OR neonat* OR infant, newborn[mh] OR child*[mh] OR children OR child, preschool[mh] OR adolesc*[mh] OR teen* OR minor* OR “young adult*” OR pediatric OR paediatric
#2 Sample	"respiration, artificial"[mh] OR "respiration, artificial" OR ventilat* OR ventilators, mechanical [mh] OR "mechanical ventilation" OR "invasive ventilation" OR "home mechanical ventilation" OR respiratory insufficiency[mh] OR respiratory artificial OR tracheostom*[mh] OR tracheotomy OR ventilat* dependen* OR "technology dependen*" OR "chronic ventilat*" OR “long-term mechanical ventilation” OR “long term ventilation” OR “respiratory support” OR “medical* complex*” OR “medically fragile” OR “chronic disease” OR “complex care” OR “complex need” OR “complex healthcare needs” OR “special healthcare needs” OR “special need” OR CYSHCN OR “children/youth with special health care needs”
#3 Sample	home* OR “home care” OR “home nursing” [mh] OR "home care services"[mh] OR “home health services” OR "home health aides"[mh] OR "nursing homes"[mh] OR "long term care"[mh] OR “long-term care” OR “medical home”
#6 Outcome (Evaluation)	readmission OR “patient readmission” OR rehospitalization* OR “unplanned readmission” OR “unplanned hospital readmission” OR “thirty day readmission” OR “30 day readmission” OR “hospital readmission” OR readmittance OR “preventable hospitalization” OR “hospital resource use” OR “health care utilization” OR “emergency room visit” OR “emergency department visit” OR mortality [mh] OR mortality [tiab] OR “child mortality” OR death
#7	Search #1 AND #2 AND #3 AND #6
**Search**	**Embase query**
#1 Sample	'child'/exp OR child OR infant OR adolescent OR 'young adult' OR pediatric OR paediatric
#2 Sample	('tracheostomy'/exp OR tracheostomy) AND ('mechanical ventilation'/exp OR 'mechanical ventilation' OR (mechanical AND ('ventilation'/exp OR ventilation)
#3 Sample	('health services'/exp OR 'health services' OR (('health'/exp OR health) AND services)
#6 Outcome (Evaluation)	('hospitalization'/exp OR hospitalization OR readmission OR rehospitalization OR 'emergency visits' OR 'healthcare utilization')
#7	Search #1 AND #2 AND #3 AND #6
**Search**	**Web of Science query**

#1 Sample	pediatric OR paediatric OR infant OR child OR children OR adolescent OR “young adults”
#2 Sample	Tracheostomy OR “mechanical ventilation" OR "invasive ventilation" OR “long term ventilation” OR “home ventilation” OR “respiratory artificial” OR “medically complex” OR “medical complex” OR “technology dependent”
#3 Sample	“home care” OR “home nursing” OR "home care services" OR “health services” OR "home health aides" OR "nursing homes" OR "long term care" OR “long-term care” OR “medical home”
#6 Outcome (Evaluation)	readmission OR “patient readmission” OR rehospitalization OR “unplanned readmission” OR “unplanned hospital readmission” OR “thirty day readmission” OR “30 day readmission” OR “hospital readmission” OR readmittance OR “preventable hospitalization” OR “hospital resource use” OR “health care utilization” OR “emergency room visit” OR “emergency department visit” OR mortality OR “child mortality” OR death
#7	Search #1 AND #2 AND #3 AND #6
**Search**	**CINAHL query**
#1 Sample	pediatric OR paediatric OR infant OR newborn OR child OR children OR adolescent OR “young adults” OR MH "Child+"
#2 Sample	"mechanical ventilation" OR "invasive ventilation" OR “long term ventilation” OR “home ventilation” OR “medically complex” OR “complex care” OR “technology dependent” OR tracheostomy OR MH "Respiration, Artificial+"
#3 Sample	“home care” OR “home nursing” OR "home care services" OR “health services” OR "home health aides" OR "nursing homes" OR "long term care" OR “long-term care” OR “medical home”
#6 Outcome (Evaluation)	readmission OR “patient readmission” OR rehospitalization* OR “unplanned readmission” OR “unplanned hospital readmission” OR “thirty day readmission” OR “30 day readmission” OR “hospital readmission” OR readmittance OR “preventable hospitalization” OR “hospital resource use” OR “health care utilization” OR “emergency room visit” OR “emergency department visit” OR MH "Mortality+" OR mortality OR "child mortality" OR death
#7	Search #1 AND #2 AND #3 AND #6
**Search**	**Epistemonikos query**
#1 Sample	pediatric OR paediatric OR child OR children OR infant
#2 Sample	mechanical ventilation OR tracheostomy

#6 Outcome (Evaluation)	readmission OR rehospitalization OR mortality
#7	Search #1 AND #2 AND #6

Table 2. Summary of Readmission and Mortality Outcome Measures

**Table 2. Summary of Outcome Measures**	**Group 1: Tracheostomy only and LTMV-T**														**Group 2: LTMV-T**										**Group 3: NIV and LTMV-T**			
	Lodge [1]	Beams [2]	Kukora [3]	Liu [4]	Van Horn [5]	Perez [6]	Phuaksaman [7]	Temur [8]	Muesing [9]	Russell [10]	Akangire [11]	Russell [12]	Yu[ ]13	Ortmann [14]	Akangire [15]	Giambra [16]	Borges [17]	Rogerson [18]	Ertugrul [19]	Henningfeld [20]	Cristea [21]	Cristea [22]	Kun [23]	Gilgoff [24]	Özcan [25]	Pavone [26]	Amin [27]	Pekcan [28]
**Readmission Outcomes**																												
Readmission ≤30 days post-discharge	✓				✓	✓	✓						✓					✓		✓			✓					
Readmission ≤1 year post-discharge												✓				✓		✓					✓					
Readmission ≤2 years post-discharge		✓																										
Readmission first 2 to 4 years of life											✓				✓													
Time to first-readmission										✓																		
Readmission during study period																	✓		✓		✓	✓		✓	✓			
**Mortality Outcomes**																												
Mortality <1 year from discharge				✓														✓			✓		✓	✓				
Mortality <5 years from discharge		✓	✓	✓			✓	✓			✓				✓						✓			✓				✓
Mortality <15 years				✓					✓					✓			✓		✓					✓				
Mortality >15 years																						✓				✓	✓	

Table 3. Factors for Readmission and Mortality

Key

Symbol	Meaning
(blank)	Not reported
+ (green)	Multivariable reported results were statistically significant
+ (light green)	Univariate reported results were statistically significant
− (orange)	Reported results were not statistically significant
↑↓	Increasing or decreasing risk of association if available

		Group 1: Tracheostomy only and LTMV-T												Group 2: LMTV-T								Group 3: NIV and LTMV-T	
		Lodge [1]	Beams [2]	Kukora [3]	Liu [4]	Van Horn [5]	Perez [6]	Phuaksaman [7]	Russell [10]	Akangire [11]	Russell [12]	Yu [13]	Ortmann [14]	Giambra [16]	Borges [17]	Rogerson [18]	Ertugrul [19]	Cristea [21]	Cristea [22]	Kun [23]	Gilgoff [24]	Özcan [25]	Pekcan [28]
**Factors for readmission**																							
Child demographics	Age													+									
Child demographics	Age at tracheostomy/ventilator (younger ages)		−			−		−		−										−		+↑ (light green)	
Child demographics	Age at admission (younger patients <5 years)	−					−		+↑		−	−											
Child demographics	Gender	−	−			−		−	−		−			−						−			
Child demographics	Race and ethnicity (Black/Hispanic)	−	+↑			−			+↑		+↑			−						−			
Child demographics	Primary language (Spanish-speaking)		+↑																				
Clinical Characteristics	Underlying diagnosis (neurologic)							−	+↑	−						−						+↑ (light green)	
Clinical Characteristics	Tracheostomy indication		−								−	−											
Clinical Characteristics	Comorbidities	−	+↑ (light green)			+↑ (light green)						+↑								−			
Clinical Characteristics	Number of chronic conditions								+↑					+									
Clinical Characteristics	All Patients Refined Diagnosis Related Groups (APR- DRG)													−									
Clinical Characteristics	Prematurity		−				−				−	+↓											
Clinical Characteristics	PH Subgroup (Secondary PH CHD only)						+↓																
Clinical Characteristics	RACHS								−														

	Respiratory cultures (pseudomonas between tracheotomy & discharge)										+↑												
	Length of stay/Post-tracheostomy length of stay	–	+↑			+↑		–			–	+↑											
	Intensive Care Unit stay													–									
	Source of admission													+									
	Discharge disposition (home)	–				–			+↑					+						–			
	Time of the year discharged								–														
	Change in management before discharge																			+↑			
	Medical device (gastrostomy tube, feeding tube)	+↑							+↑		–	+↓					+↑			–			
	Medications or treatments (inhaled steroids; pro-gut motility agents)						–			+↑	+↓												
	Ventilator or oxygen dependence	–	+↑			–	–	–	+↓	+↑	–	+↑										+↑	
	Type of tracheostomy/ventilator							–												–			
	Hours on ventilator																			–			
	Hospital region													–									
	Clinic visit post-discharge																			–			
	Duration of caregiver education (<14 days)																					+↑	
	Invasive procedures at index hospitalization (CPR, ECMO)						–																
Socioenvironmental	Income/poverty		–															–					
	Insurance type (public)	–	–			+↑			+↑		–	–		+						–			
	Home care services																			–			
	Number of equipment malfunctions									+↑													

	Rural vs. urban living (rural)					+↓																	
	Quality of life		–																				
	Area Deprivation Index		–																				
**Risk factors for mortality**																							
Child demographics	Gestational age			–	–														–				
Child demographics	Age at tracheostomy/ventilator			–																			
Child demographics	Age at admission														–								
Child demographics	Gender			–	+↑														–				
Child demographics	Race and ethnicity			–	–														–				
Clinical Characteristics	Birth weight (≤750 grams)			–	–														+↑				
Clinical Characteristics	Underlying diagnosis														–	–					–		
Clinical Characteristics	Cardiac diagnosis (congenital heart disease)				+↑								–										
Clinical Characteristics	Tracheostomy indication (airway obstruction)			+↑	–								+↓										
Clinical Characteristics	Comorbidities (severe neurologic disability)			–	+↑														–				
Clinical Characteristics	Length of stay																		–				
Clinical Characteristics	Discharge disposition																		–				
Clinical Characteristics	Readmission														+↑								
Clinical Characteristics	Medical device (any tube feeding)			+↑																			
Clinical Characteristics	Medications or treatments (physiotherapy)			–													+↓						
Clinical Characteristics	Ventilator or oxygen dependence			–	+↑								–										

Socioenvironmental	Income/poverty (lower median household income)																	+↑					
	Insurance type				–														–				
	Maternal education level																						–
	Rural vs. urban living																						–

Footnote: PH=pulmonary hypertension; RACHS=risk adjustment for congenital heart surgery score; CPR=cardiopulmonary resuscitation; ECMO=extracorporeal membrane oxygenation.

Table 4. Summary of Readmission Measures and Results (N=20 studies)

	First author/ year published/ Country	Findings	Outcomes/ Length of follow-up	Readmission					
				Total # of readmissions n(%)	No. of children who experienced readmission n(%)	Average no. of readmissions<br><br>Median(IQR)<br>Mean(±SD)	Reason for readmission n(%)	Readmission rate/ readmission length of stay	Time to readmission
**Group 1: Tracheostomy *and* LTMV-T population**<br>*Footnote*: results for this section are split based on overall sample (OS), LTMV via tracheostomy (LTMV-T), and tracheostomy only (TO) results									
Readmission within 30-days from discharge	#1 Lodge/ 2024/ United States	Among children with LTMV-T, 30-day all-cause readmission was 14% and respiratory-related readmission was 5.8%. For children on LTMV-T, there was no association between discharge with home health nursing and 30-day all cause (aOR=1.11, 95% CI: 0.30-4.02) or respiratory-related readmission (aOR=3.68, 95% CI: 0.38-35.60). Trach/gastrostomy tube placement increased odds of 30-day all-cause readmission (aOR=4.41, 95% CI: 1.08-17.97). Insurance and neuromuscular disease were not significant for 30-day readmission. Insurance, neuromuscular disease, trach/gastrostomy tube placement was not associated with respiratory-related readmission.	30-day all-cause same-hospital and respiratory-related readmission	NR	19(14.6) OS, 30-day all-cause<br><br>12(14) LTMV-T, 30-day all-cause<br>5(5.8) LTMV-T, respiratory-related<br><br>7(15.9) TO, 30-day all-cause<br>4(9.1) TO, respiratory-related	NR	NR	NR	NR

	#5 Van Horn[a]/ 2023/ United States	All-cause readmission was 28% for both the overall sample and children on LTMV-T. For the overall sample, odds of 30-day readmission was lower in rural patients (aOR= 0.80, 95% CI: 0.68-0.95) and higher in publicly insured (aOR=1.24, 95% CI: 1.09-1.42). Odds of tracheostomy-related admission were higher in publicly insured children (aOR=1.39, 95% CI: 1.03-1.88). No difference in odds of tracheostomy-related admission by rurality.	All-cause 30-day readmission following discharge from the index hospitalization	NR	1859(28) OS<br>936(28) LTMV-T<br>NR TO	NR	321/1859 (17) Tracheostomy-related diagnosis<br>141/936(15) tracheostomy-related diagnosis<br>NR	NR	NR
	#6 Perez[a]/ 2022/ United States	33.3% of children had at least one readmission within 30 days of index discharge or subsequent encounters during the 2-year follow-up period. Among children discharged on LTMV-T, 31% had a 30-day hospital readmission. For the overall sample, secondary PH due to CHD (aOR= 0.43, 95% CI: 0.24–0.77) and tracheostomy placement during the index encounter (aOR= 0.34, 95% CI: 0.19–0.61) were associated	30-day readmission following discharge from the index hospitalization	NR	216(33.3) OS	NR	53/176(30.1) Respiratory failure<br>34(19.3) Infection/sepsis<br>28(15.9) Tracheitis and/or pneumonia<br>12(6.8) Equipment malfunction<br>11(6.2) Disorders of the cardiac system<br>10(5.7) Disorders of the gastrointestinal tract<br>7(4.0) Pulmonary hypertension<br>21(11.9) Other (pleural effusion, airway anomalies, medication effects, and disorders of the neurological, hematological, renal systems)	NR	NR

		with fewer 30-day readmissions.			97(31%) LTMV-T		NR		
					NR TO		NR		
	#7 Phuaksaman / 2022/ Thailand	17.6% of the overall sample and 9.5% of children on LTMV-T had an unplanned readmission for acute respiratory distress within 30 days. Pneumonia was the primary reason for readmission for the overall sample and no risk factors (age, age at tracheostomy, time to tracheostomy, underlying disease, or trach/ventilator type) were associated with 30-day unplanned readmissions.	30-day unplanned readmissions	NR	15(17.6) OS, unplanned readmission for acute respiratory distress	NR	9(60) Pneumonia<br>6(40) Tracheitis	NR	NR
					2(9.5%) LTMV-T, unplanned readmission for acute respiratory distress		NR		
					13(20%) TO, unplanned readmission for acute respiratory distress		NR		
	#13 Yu[b]/ 2017/ United States	Over 1/5 of patients in this cohort were readmitted 30 days from discharge. Children on LTMV-T had 24% readmitted. 92% of readmissions were unplanned. Among the overall sample of children ≤12 months, those with prematurity (aOR=0.35, 95% CI: 0.15 to 0.83) and gastrostomy tube placement (aOR=0.42,	30-day all-cause hospital readmission	NR	60(22) OS	NR	35(64) Respiratory infections<br>11(20) Gastrointestinal diagnoses<br>3(5.5) Tracheostomy complications<br>5(9) Non-respiratory infectious diagnoses	NR	NR
					31(24) LTMV-T		NR		

		95% CI: 0.19-0.96) had decreased odds of 30-day readmission. Discharge with LTMV-T was associated with increased odds (aOR=2.88, 95% CI: 1.19-6.97). Among children >1 year of age, increased LOS (aOR=1.01 per day, 95% CI: 1.00-1.02) and comorbid malignancy (aOR=6.03, 95% CI: 1.25-29.16) increased the odds of readmission.			NR TO		NR		
Readmission within 12 months from discharge	#12 Russell[b]/ 2017/ United States	43% of children had a bTARTI readmission within 12 months. Of the children who were on LTMV-T, 37% readmitted. The primary reason for readmission was bacterial tracheitis (39%). Among the overall sample, Hispanic ethnicity (aOR=2.0, 95% CI: 1.06–3.85) and acquisition of Pseudomonas aeruginosa between tracheotomy and discharge (aOR=3.2, 95% CI: 1.23–8.31) were associated with increased odds of bTARTI readmission. Gastrointestinal pro-motility agents were associated with decreased risk (aOR=0.43, 95% CI: 0.23–0.8).	Readmitted for bacterial tracheostomy-associated respiratory tract infection (bTARTI) within 12 months of discharge post-tracheostomy	NR	103(43) OS	NR	40(39) bacterial tracheitis<br>35(34) bacterial pneumonia<br>28(27) both bacterial tracheitis and pneumonia	NR	NR
					41(37) LTMV-T		NR		
					NR		NR		

Readmission within the first 2 years after tracheostomy	#2 Beams/ 2023/ United States	Among children on LTMV-T 52% had >4 visits mostly occurring in the first year from discharge. Among the overall sample, Black race (OR=2.01, 95% CI: 1.18–3.70), mechanical ventilation (OR=2.74, 95% CI: 1.35–5.59), and Spanish-speaking (OR=3.86, 95% CI: 1.47–10.11) children had the highest utilization. Hispanic ethnicity (OR=1.57, 95% CI: 1.22–2.02) and gestational age (OR=0.94, 95% CI: 0.93–0.97) were associated with increased respiratory-related revisits.	Frequent visits defined as 4 or more emergency department (ED) and/or hospitalization encounters during the 24-month study period after tracheotomy hospitalization	Overall sample:<br>1285 Total ED and/or hospital visits<br><br>870/1285(68) hospital visits<br><br>415/1285(32) ED visits	Overall sample:<br>127(53) <4 visits<br><br>112(47) ≥4 visits	7(NR) median encounters	28(2.2) Gastroenteritis<br>20(1.6) Sepsis<br>73(5.7) Upper respiratory infection<br>62(4.8) Pneumonia/<br>14(1.1) Congenital heart disease<br>62(4.8) Tracheostomy-related<br>119(9.3) Gastrostomy-related<br>38(3.0) Seizures<br>471(37) Acute/chronic respiratory failure	NR	65% of visits occurred in the first year<br><br>40% of visits occurred in the first 6 months
				LTMV-T:<br>191 total ED or hospital visits<br><br>1105 total respiratory-related ED or hospital visits	LTMV-T:<br>92(48%)<4 visits<br><br>99(52%) >4 visits	NR	616/1105 (56) Respiratory<br>489/1105 (44) Non-respiratory	NR	NR
				NR TO	NR TO	NR	NR	NR	NR
Readmission within first 2 years of life	#11 Akangire/ 2017/ Canada	Unplanned or elective readmissions primarily occur within the first 1.5 years of life. 40% of readmissions were before 1 year of age. Viral infections were the most common reason for admission. Among the overall sample, ventilator dependence at 6 months of age (aOR=29.2, 95% CI 1.99-429.44) and 12 months (aOR=20.8, 95% CI 1.28-337.41) of life was associated with readmission. Age at	Number of rehospitalizations between 0 and 12 months of age, number of rehospitalizations between 13 and 24 months of age	NR(54) from discharge to 12 months of age<br><br>NR(53) from 12-18 months of age<br><br>NR(28) from 18-24 months of age	NR	NR	39(24) Pulmonary infections<br>36(22) Other pulmonary<br>35(21) Other<br>23(14) GI and feeding issues<br>13(8) Equipment malfunction (tracheal plugdislodgement, ventilator/tubing malfunction, obstruction from granuloma, g-tube dislodgement)<br>13(8) Surgery<br>1(0.6) Cardiac	NR	NR
					38(70) LTMV-T		NR		

		tracheostomy, ventilator at discharge, diuretics, inhaled steroids, dysphagia, and pulmonary hypertension were not significant in the multivariate model. Equipment malfunctions increased risk of readmission (OR=8.50, 95% CI: 2.63-∞).			23(50) TO		NR		
Time to first readmission	#10 Russell[a]/ 2018/ United States	36% of the overall sample and 39% of those on LTMV-T experienced at least one bacterial respiratory tract infection (bRTI) admission. Among the overall sample, younger age (age < 30 days vs 13-17 years [aHR=1.32, 95%CI: 1.11-1.58]), Hispanic race/ethnicity (aHR= 1.34, 95% CI: 1.20-1.50), government insurance (aHR=1.21, 95%CI: 1.1-1.33), neurologic comorbidity (aHR=1.29; 95% CI: 1.19-1.40), g-tube dependence (aHR=1.15; 95% CI: 1.05-1.26), >2 complex chronic conditions (aHR=1.96, 95%CI: 1.34-2.86) and discharge to home (aHR 1.19, 95%CI: 1.08-1.32) were associated with increased risk of bRTI readmission. Trauma diagnosis (aHR=0.83, 95%CI: 0.69-1.00) and	First bRTI readmission after tracheostomy	NR	2899(36) OS at least one bRTI admission<br>704(39) LTMV-T<br>NR TO	NR	100% bacterial respiratory tract infection	NR	275 days (IQR 141-530) median time to first bRTI readmission<br>NR<br>NR

		ventilator dependency (aHR=0.88, 95%CI: 0.81-0.97) were associated with decreased risk.							
**Group 2: LTMV-T population**									
Readmission within 30 days	#20 Henningfeld/ 2016/ United States	Within 30 days from discharge, 35% of patients were readmitted. A median of 5 readmissions occurred prior to decannulation. Respiratory infections were the primary cause of readmission.	Readmission within 7 and 30 days from discharge	NR	7(15) within 7 days<br>16(35) within 30 days	5 median readmissions	45 Respiratory infection<br>22 Non-respiratory illness<br>20 Surgery<br>8 Social<br>2 Ventilator management	3 median days	NR
Readmission within 12 months	#18 Rogerson/ 2020/ United States	More than 75% of children had a readmission within the first year from discharge. Children with a neurological disorder had fewer average hospital-free days (269) compared to those with a respiratory disorder (319), however these differences were not statistically significant (p=0.07).	Readmission within the first year after discharge	NR	19(38) First month<br>27(54) First 3 months<br>38(76) First 12 months	2.16 mean readmissions per subject in the first year after discharge	NR	NR	NR

	#16 Giambra[a]/ 2021/ United States	Children with established LTMV-T had a median number of 2 readmissions within 365 days from index hospitalization discharge. Age, insurance type, admission source, disposition, and number of complex chronic conditions were associated with readmissions. Gender, race, region, and ICU stay, and All Patients Refined Diagnosis Related Groups were not statistically significant.	Readmission within the 365 days following their hospitalization	NR	NR	2(IQR 1-4) median number of readmissions	NR	NR	NR
	#23 Kun/ 2012/ United States	78 readmissions occurred within the first year of discharge. Majority (64%) of readmissions were due to pulmonary or tracheostomy-related problems. Change in patient management within 7 days of discharge was associated with readmission in the univariate analysis (18% readmitted vs. 3% not readmitted, p=0.01). Age, epilepsy, place of residence, ventilator type, feeding tube, and neurodevelopmental delay were not significant in the multivariate model.	Readmission within the first year from discharge	78 readmissions	19(17) 1 month<br>22(20) 3 months<br>44(40) 12 months	0.7(SD ±1.1; range 0-5), mean number of non-elective readmissions per patient<br><br>Among the readmitted patients, mean number of readmissions per patient was 1.9 (SD ± 1)	22(28) Pneumonia<br>13(17) Tracheitis<br>9(11.5) Tracheostomy decannulation/obstruction<br>4(5) Tracheostomy bleeding<br>2(2.5) Respiratory, other<br>5(6.5) Infectious, other<br>8(10) Abdominal pain/emesis<br>4(5) Gastrointestinal, other<br>3(4) Failure to thrive/feeding intolerance<br>3(4) Neurosurgical<br>2(2.5) Seizures<br>2(2.5) Dehydration/ electrolyte imbalance<br>1(1.5) Ophthalmologic	11(IQR 8-25) median length of stay in days among those with a readmission	NR

Readmission within first 4 years of [illegible]	#15 Akangire/ 2023/ United States	Of the 98 infants, 83 (84.6%) required rehospitalizations, 67.4% had 1–4 rehospitalization events, and 15.7% had >4 events. Majority of rehospitalizations occurred in the first 24 months of age (75.8%). Respiratory system problems were the primary reason for rehospitalization (79.6%).	Rehospitalization from the neonatal intensive care unit discharge to 12 months of age, 13 to 24 months of age, and 25 to 36 months of age.	85 from NICU discharge to 12 months of age<br>112 from 13-24 months<br>63 from 25-36 months	83(84.6) rehospitalization<br>15(15.3) had no rehospitalization<br><br>56(67.4) had 1-4 visits<br>13(15.7) had >4 visits	NR	207 (79.6) respiratory system<br>53 (20.4) non-respiratory	2(1,5) median(IQR) length of stay in days	NR
Readmission during study period	#17 Borges/ 2020/ Brazil	Mean duration of days at home (995.0(SD±4.6)) was higher than the mean days in the hospital (341(SD±0.5)). Respiratory tract infections (48.3%) are the primary cause of readmission. Among respiratory tract infections, tracheitis was the major cause of readmission (66.5%).	Days free from hospitalization during the 10-year study period	NR	NR	NR	48.3% Respiratory tract infection<br>13.3% Device failure<br>13.3% Urinary tract infection<br>11.7% Other<br>5.0% Gastrointestinal disturbances<br>5.0% Myopathies<br>3.3% Metabolic disturbances	NR	392.6 (SD± 548.9) mean days to first hospital readmission
	#19 Ertugrul/ 2017/ Turkey	37% of children experienced a readmission. Pneumonia was the primary cause for readmission. Rehospitalizations were observed more frequently in patients with gastrostomy tubes compared to nasogastric tubes ($p<0.05$).	Readmission during study period	45 occasions	23(37%)	NR	34(75) Pneumonia<br>3 Dehydration<br>1 Sepsis<br>1 Fundoplication<br>1 Renal Failure<br>1 AED Induced Thrombocytopenia<br>2 MV Repair<br>1 Scoliosis Surgery<br>1 Peritonitis	NR	NR

	#21 Cristea[c]/ 2015/ United States	The median hospitalization rates/year with a respiratory diagnosis among children with zip code-based annual household income (Z-AHI) below the median state household was 0.92 vs. those with Z-AHI above the median state household was 0.55 however this was not statistically significant (p=0.75). Non-respiratory hospitalizations were also not significantly different between Z-AHI below and Z-AHI above the median state household (0.26 vs. 0.21, p=0.94).	Hospitalization rates per year from discharge	NR	NR	NR	NR	Z-AHI below median state household: 0.92(0-60) median (range) respiratory hospitalization rates/year 0.26(0-4.8) median (range) non-respiratory<br><br>Z-AHI above median state household: 0.55(0-16) median (range) respiratory hospitalization rates/year 0.21(0-2.57) median (range) non-respiratory	NR
	#22 Cristea[c]/ 2013/ United States	There was a total of 554 rehospitalization events before decannulation in the 27-year study period with a higher rate before decannulation vs. after. The primary reason for readmission was respiratory related (65%).	Number of rehospitalizations after index discharge	554 rehospitalization events	NR	NR	358(64.6) Respiratory related<br>54(9.7) Surgical interventions<br>142(25.6) NR	NR	NR
	#24 Gilgoff/ 2003/ United States	87% of children required a rehospitalization during the 20-year study period. Children with neuromuscular diseases had more rehospitalizations 0.8/year (range, 0-2.14) compared to children with spinal cord injuries 0.6/year (range, 0-1.46).	Readmissions within 20 years from index hospitalization	NR	27(87)	0.7(0-2.14) mean(range) per year	NR(60) Pneumonia<br>NR(39) Scheduled surgeries<br>4(13) Seizure disorders	NR	NR

Group 3: Non-invasive mechanical ventilation and LTMV-T **populations**

Footnote: results for this section are split based on overall sample (OS), LTMV via tracheostomy (LTMV-T), and non-invasive mechanical ventilation (NIV) results

Readmission during study period	#25 Özcan/ 2021/ Turkey	60% of children on LTMV-T were readmitted. Among the overall sample, invasive ventilation (vs. non-invasive; OR=16.3, 95% CI 2.1–127.4), caregivers who spent <14 days in the hospital for education (vs. >14 days; OR=4.0, 95% CI 1.5–11.2), younger age of 0-24 months (vs. >60 months; OR=3.25, 95% CI: 1.07-9.91), and neurologic/muscular disease (vs. airway; OR=9.26, 95% CI: 1.14-74.70) increased risk of readmission.	Nonscheduled hospital readmissions after discharge during a 6-year study period	NR	29(29.8) OS	NR	9(31) Tracheostomy-related complications<br>5(17.2) Acute lower respiratory tract infection<br>6(20.7) Seizures<br>5(17.2) Feeding problems<br>3(10.3) Infection<br>1(3.4) Dehydration/ electrolyte imbalance	NR	18.1 ± 11.6 mean days from discharge to first readmission
					42(60) LTMV-T		NR		NR
					1(4) NIV		NR		NR
	#27 Amin/ 2014/ Canada	Children on LTMV-T had a greater median number of emergency visits/year compared to NIV (0.3 vs. 0, p=0.003). The number of days in the hospital/year was also greater among children on LTMV-T compared to NIV (13.2 vs. 2.4, p=0.002).	Number of emergency visits and days in hospital per year for 20 years	NR	NR	0 (0–0.7) emergency visits/year OS	NR	NR	NR
						0.3(0-1.1) emergency visits/year LTMV-T			
						0 (0–0.7) emergency visits/year NIV			

Footnote: [a]Studies that used the same Pediatric Health Information System (PHIS) national database
[b]Reports that use the same data/population from Children's Hospital Los Angeles and therefore count as one study
[c]Reports that use the same data/population from Riley Hospital for Children and therefore count as one study

Table 5. Summary of Mortality Measures and Results (N=17)

First author/ year published/ Country	Findings	Outcome/ length of follow-up	Mortality					
			No. died (%)	Mortality rate / survival rate	Median age at death (IQR)	Cause of death	Location of death No. (%)	Time to death
**Group 1: Tracheostomy *and* LTMV-T population**<br>*Footnote*: results for this section are split based on overall sample (OS), LTMV via tracheostomy (LTMV-T), and tracheostomy only (TO) results								
#3 Kukora/ 2023/ United States	Mortality after discharge for children on LTMV-T was 20%. For the overall sample, airway obstruction as an indication for tracheostomy (aOR=1.53, 95% CI: 1.04-2.26) increased risk of mortality at home and tube feeding was associated with increased risk of mortality (aOR=1.14, 95% CI: 1.00-1.31). Tube feeding was no longer significant after excluding patients who discharged on hospice/palliative care. Birthweight, gender, any anomaly, and type of pulmonary support were not significant.	Three-year survival up to 6 years starting from the date of tracheostomy placement	40(18) OS	82% alive at 3 years after tracheostomy	479(252–899) days of life	7 Cardiac/respiratory arrest<br>6 Sepsis/pneumonia<br>3 Seizure-related complications<br>2 Suspected abuse or neglect<br>3 Surgical/medical complication<br>1 Ventilator malfunction causing pneumothorax<br>4 Palliative care<br>1 Hospice<br>1 Tracheotomy complications<br>5 Unknown	15(38) Home<br>25(63) Hospital	252(55–656) duration discharge to death, days
			26(20) LTMV-T	NR	519(293–945) days of life	16 Cardiac/respiratory arrest<br>5 Sepsis/pneumonia<br>1 Seizure-related complications<br>1 Suspected abuse/neglect<br>3 Medical/surgical therapy complications<br>1 Ventilator malfunction	9 Home<br>17 Hospital	252(48–692) duration discharge to death, days
			14(14) TO	NR	370 (153–970) days of life	9 Cardiac/respiratory arrest<br>1 Sepsis/pneumonia<br>2 Seizure-related complications<br>1 Suspected abuse/neglect	6 Home<br>8 Hospital	252(58–681) duration discharge to death, days
#4 Liu/ 2023/ United States	22% of children on LTMV-T died by the end of the study. Ventilator at index discharge (aHR=2.04, 95% CI: 1.10-3.81), severe neurologic disability (aHR=2.79,	1-, 5-, and 10-year survival rates	113(21) OS	11.9% (95% CI 9.4-15.1) 1-year cumulative mortality<br><br>26.1% (95% CI 21.6-31.3) 5-year	NR	NR	NR	NR

	95% CI: 1.61, -4.84), and congenital cardiac disease (aHR=1.69, 95% CI: 1.08-2.65) were associated with time to death. Female sex was significant in the univariate analysis (HR=1.47, 95% CI: 1.01-2.12). Age, race, insurance, sepsis, trauma, respiratory failure, and congenital malformations were not significant.			cumulative mortality<br><br>41.6% (95% CI 32.7-51.8) 10-year cumulative mortality				
			79(22) LTMV-T	11.0% (95% CI 8.0-14.9) 1-year cumulative mortality<br><br>27.9% (95% CI 22.2-34.6) 5-year cumulative mortality<br><br>43.7% (95% CI 33.8-55.2) 10-year cumulative mortality				
			NR TO	NR				
#7 Phuaksaman/ 2022/ Thailand	Among the overall sample and LTMV-T group, 7% and 14% died respectively. The primary cause of death was underlying disease progression.	Mortality during study follow-up period (2012-2020)	6(7) OS	NR	2.1±1.3 years (range 9 months-3.3 years) mean age	1 Pneumonia<br>2 Septic shock<br>3 Primary diseases (i.e. Loeys Dietz syndrome, Jansen-type metaphyseal chondroplasia, post heart transplantation for fulminant myocarditis)	NR	1.6±1.5 years (range 3 months-3.1 years) mean time interval between tracheostomy and death
			3(14) LTMV-T			NR		
			3(4.7) TO			NR		
	Among children who received congenital	Mortality during 4-year study	14(31.1) OS	NR	NR	NR	7 Home<br>6 Hospital	NR

#8 Temur/ 2021/ Istanbul	heart surgery and LTMV-T, 41% died during the mean follow-up time of 36 months. There was no statistically significant difference between operation status (i.e., total repair vs. palliation operation) and mortality (p=0.48).	period (2014-2018)	13(41) LTMV-T					
			NR TO					
#9 Muesing/ 2020/ United States	The all-cause mortality rate from 2008-2018 was 13.11% for LTMV-T. Longitudinal trends showed a statistically significant 0.5 percentage point decline in annual all-cause death rate over the 11-year period from 8.8% to 3.3% for children on LTMV-T. There was no significant difference in overall mortality rate across age groups.	Annual all-cause death rate	4.5% death rate OS	13% all-cause mortality rate during 2008-2018<br><br>8.8% annual all-cause death rate in 2008<br>3.3% annual all-cause death rate in 2018<br><br>83% 5-year survival rates<br>60% 10-year survival rates	NR	NR	NR	NR
			4.6% death rate LTMV-T	13.11% all-cause mortality rate during 2008-2018				
			4.4% death rate TO	12.73% all-cause mortality rate during 2008-2018				
#11 Akangire/ 2017/ Canada	Among the overall sample, 4% of infants died by 2 years of age.	Mortality after discharge for first 2 years of life	4(4) OS	NR	NR	NR	NR	NR

#14 Ortmann/ 2017/ United States	At a median follow-up of 3.9 years, 33% of those who survived to discharge died. Most deaths (92%) occurred during the first 2 years after tracheostomy. Children with upper or lower airway obstruction as the sole indication for tracheostomy had better survival compared to other indications (76% vs. 31%, p=0.003). Intermediate survival was lower in those with heart failure (28% vs 61%, p=0.029). Intermediate survival did not differ by ventilator dependence.	Hospital survival and intermediate survival, which was defined as survival as of May 31, 2015	11(33%) OS	48% intermediate survival	NR	3(27) Possible/confirmed sepsis<br>1(9) Heart failure<br>5(45) Tracheostomy tube dislodgement/obstruction<br>1(9) Later surgical complication<br>1(9) Unknown	NR	NR
			NR LTMV-T	67% intermediate survival of those who survived to discharge		NR		
			NR TO	67% intermediate survival of those who survived to discharge		NR		
**Group 2: LTMV-T**								
#15 Akangire/ 2023/ United States	Only 1 infant died from accidental decannulation in the home.	Overall survival rate calculated at a maximum age of 4 years	1(1.0)	99% survival rate	18 months (NR)	1(100) Accidental decannulation	1(100) Home	NR
#17 Borges/ 2020/ Brazil	During the 10-year study period, overall mortality was 48%. Children readmitted within <6 months of discharge had a 10% greater chance of death (OR=0.10, 95% CI: 0.02-0.65). Underlying diagnoses (i.e., cerebral palsy, genetic syndrome, neuromuscular disease, pulmonary	Mortality during the 10-year study period.	13(48%)	NR	NR	NR	10(76.9) Hospital<br>3(23.1) Home	NR

	disease) and admission age were not statistically significant risks for mortality.							
#18 Rogerson/ 2020/ United States	One year mortality rate was 16%. Mortality rates were higher among those with a neurologic disorder (29%) compared to respiratory (12%, p=0.21).	Mortality within the first year from discharge	8(16)	NR	NR	3(38) Compassionate withdrawals of support<br>2(25) Tracheostomy tube dislodgement<br>2(25)Cardiac arrests at home<br>1(12) Stroke	NR	NR
#19 Ertugrul/ 2017/ Turkey	Overall mortality rate was 46.7% with lower mortality rates among patients who received physiotherapy compared to those who did not (51% vs. 80%, p < 0.01).	Mortality during study period	29(46.7)	NR	NR	22(75) Primary disease<br>7(15) tracheostomy related complications	NR	NR
#21 Cristea[c]/ 2015/ United States	15.9% of the cohort died. Of the patients who died, 14/15 were from the zip code-based annual household income (Z-AHI) below the median state household compared to those from Z-AHI above the median state household (24% vs. 3%, p=0.003). Difference in survival probability at 60 months between the two income groups was not statistically significant.	Percentage of survival probability at 60 months of age	15(15.9)	Survival probability at 60 months of age:<br><br>81.0% (95% CI: 70.9-91.1) Z-AHI below median state household<br><br>100% (95% CI: 100.0-90.3) Z-AHI above median state household	28.4 (15.7-60) months	1 Tracheostomy related<br>2 Expected (Do not resuscitate orders)<br>2 Secondary to cardiorespiratory arrest	NR	NR
#22 Cristea[c]/ 2013/	18.6% of children died during the 27-year study period. Patients with a lower birth	Mortality after discharge in a 27-year period	19(18.6)	NR	27(16-60) months	2 Tracheostomy related<br>2 Expected (Do not resuscitate orders)	NR	NR

United States	weight (≤750 vs. >750 grams) had greater odds of an earlier death (aOR=0.34, 95% CI 0.12-0.99). Race, gender, and intraventricular hemorrhage were not associated with death.					2 Secondary to cardiorespiratory arrest		
#23 Kun/ 2012/ United States	There were 0 deaths during the first year after discharge with tracheostomy and ventilation.	Mortality after discharge	0(0%)	N/A	N/A	N/A	N/A	N/A
#24 Gilgoff/ 2003/ United States	18% of children died during the study period with 25% related to ventilator problems. There was no statistically significant difference in survival rates between neuromuscular and spinal cord injury groups (p>0.8).	Survival data starting point was the date of discharge from the hospital. End point was the date at the end of data collection (June 2001), date ventilator support was discontinued, or patient's death.	8(18)	97% at 1 year<br>97% at 3 years<br>84% at 5 years<br>71% at 10 years	11(5-18.83) mean(range) years	2(25) Ventilator-related problems<br>1(12.5) Bowel obstruction<br>1(12.5) Seizures, metabolic<br>1(12.5) Fall-accident in wheelchair<br>1(12.5) Viral illness<br>2(25) Unknown	4(50) Home<br>1(12.5) Group home<br>3(37.5)Hospital	NR
**Group 3: Non-invasive mechanical ventilation and LTMV-T populations**<br>*Footnote*: results for this section are split based on overall sample (OS), LTMV via tracheostomy (LTMV-T), and non-invasive mechanical ventilation (NIV) results								
#26 Pavone/ 2020/ Italy	14.5% of children on LTMV-T died due to the progression of neurological disorders. Compared to children on NIV, those on LTMV-T with central nervous system diseases (p=0.03) and receiving ventilation for ≥12h/day (p=0.01) died more often.	Mortality after discharge during the 17-year study period	62(14.3) OS<br>17(14.5) LTMV-T<br>45(14.3) NIV	NR	NR	NR	NR	NR

#27 Amin/ 2014/ Canada	Children on LTMV-T had a higher rate of mortality (30%) compared to non-invasive (11%) during the study period (p=0.0002). Majority of deaths occurred in the hospital. LTMV-T deaths were predominantly due to respiratory failure, accidental decannulation, and sepsis.	Mortality after discharge during the 20-year study period	55(15) OS	NR	5.03 years (IQR 1.5–13.6)	22(40) Respiratory failure<br>6(11) Cardiac<br>1(2) Aspiration<br>5(9) Accidental mask removal/ decannulation<br>4(7) Sepsis<br>1(2) Gastrointestinal bleed<br>2(4) Hypoxic ischemic encephalopathy<br>1(2) Dehydration<br>13(24) Unknown	20(36) Intensive care unit<br>4(7) Hospital ward<br>7(13) Local hospital<br>19(35) Home<br>5(9) Unknown	NR
			20(30) LTMV-T		NR	5(25) Respiratory failure<br>2(10) Cardiac<br>4(20) Accidental decannulation<br>4(20) Sepsis<br>1(5) Gastrointestinal bleed<br>1(5) Dehydration<br>3(15) Unknown	NR	
			35(11) NIV		NR	17(49) Respiratory failure<br>4(11) Cardiac<br>1(3) Accidental mask removal<br>1(3) Aspiration<br>2(6) Hypoxic ischemic encephalopathy<br>10(29) Unknown	NR	
#28 Pekcan/ 2010/ Turkey	21% of children on LTMV-T died during the 4-year study period. Death occurred within the first 7.5 months following discharge. There were no significant differences in mortality between maternal education levels (primary school vs. high school, p>0.05) or distance from the hospital (rural vs. urban, p>0.05).	Mortality within 4-year study period	11(41) OS	NR	NR	NR	7(64) Home<br>4(36) Hospital	NR
			3(21) LTMV-T					
			8(62) NIV					

Footnote: NR= not reported; [c]Reports that use the same data/population from Riley Hospital for Children and therefore count as one study
